# Assessment of the *ptxD* gene as a growth and selective marker in *Trichoderma atroviride* using P*ccg6*, a novel constitutive promoter

**DOI:** 10.1186/s12934-020-01326-z

**Published:** 2020-03-18

**Authors:** Nohemí Carreras-Villaseñor, José Guillermo Rico-Ruiz, Ricardo A. Chávez Montes, Lenin Yong-Villalobos, José Fabricio López-Hernández, Pedro Martínez-Hernández, Luis Herrera-Estrella, Alfredo Herrera-Estrella, Damar López-Arredondo

**Affiliations:** 1 StelaGenomics México, S de RL de CV, Av. Camino Real de Guanajuato s/n, 36821 Irapuato, Guanajuato Mexico; 2 Present Address: Laboratorio Nacional de Genómica para la Biodiversidad, Unidad de Genómica Avanzada del Centro de Investigación y de Estudios Avanzados del Instituto Politécnico Nacional, Km 9.6 carretera Irapuato León, 36500 Irapuato, Guanajuato Mexico; 3 Institute of Genomics for Crop Abiotic Stress Tolerance, Texas Tech University, Lubbock, TX 79409 USA; 4 Present Address: Red de Estudios Moleculares Avanzados, Instituto de Ecología A.C, Xalapa, 91070 Mexico; 5 Present Address: Stowers Institute for Medical Research, Kansas City, MO 64110 USA

**Keywords:** *ccg6* promoter, Phosphite oxidoreductase (PTXD), Phosphite, Growth marker, Contamination control

## Abstract

**Background:**

*Trichoderma* species are among the most effective cell factories to produce recombinant proteins, whose productivity relies on the molecular toolkit and promoters available for the expression of the target protein. Although inducible promoter systems have been developed for producing recombinant proteins in *Trichoderma*, constitutive promoters are often a desirable alternative. Constitutive promoters are simple to use, do not require external stimuli or chemical inducers to be activated, and lead to purer enzyme preparations. Moreover, most of the promoters for homologous and heterologous expression reported in *Trichoderma* have been commonly evaluated by directly assessing production of industrial enzymes, requiring optimization of laborious protocols.

**Results:**

Here we report the identification of P*ccg6*, a novel *Trichoderma atroviride* constitutive promoter, that has similar transcriptional strength as that of the commonly used *pki1* promoter. P*ccg6* displayed conserved arrangements of transcription factor binding sites between promoter sequences of *Trichoderma ccg6* orthologues genes, potentially involved in their regulatory properties. The predicted *ccg6*-encoded protein potentially belongs to the SPE1/SPI1 protein family and shares high identity with CCG6 orthologue sequences from other fungal species including *Trichoderma reesei*, *Trichoderma virens*, *Trichoderma asperellum*, and to a lesser extent to that of *Neurospora crassa*. We also report the use of the P*ccg6* promoter to drive the expression of PTXD, a phosphite oxidoreductase of bacterial origin, which allowed *T. atroviride* to utilize phosphite as a sole source of phosphorus. We propose *ptxD* as a growth reporter gene that allows real-time comparison of the functionality of different promoters by monitoring growth of *Trichoderma* transgenic lines and enzymatic activity of PTXD. Finally, we show that constitutive expression of *ptxD* provided *T. atroviride* a competitive advantage to outgrow bacterial contaminants when supplied with phosphite as a sole source of phosphorus.

**Conclusions:**

A new constitutive promoter, *ccg6*, for expression of homologous and heterologous proteins has been identified and tested in *T. atroviride* to express PTXD, which resulted in an effective and visible phenotype to evaluate transcriptional activity of sequence promoters. Use of PTXD as a growth marker holds great potential for assessing activity of other promoters and for biotechnological applications as a contamination control system.

## Background

*Trichoderma* is a fungal genus with high level of genetic diversity and great adaptability to different environmental conditions. These characteristics, together with factors such as their high capacity to produce and secrete proteins and bioactive compounds, as well as the availability of protocols and molecular tools for their genetic manipulation, have posed *Trichoderma* as a model organism to study fungal biology and to develop biotechnological applications for different industries [1].

Some examples of the industrial applications of *Trichoderma* are in the processing of textiles, food and feed, pulp and paper, and for the production of hydrolytic enzymes, biochemicals and antibiotics, and biofuels [1–4]. Special interest has been focused on the use of *Trichoderma* and other filamentous fungi for the expression of homologous and heterologous proteins and for the production of fungal metabolites of industrial interest. For instance, hydrolytic enzymes such as cellulases, chitinases, and xylanases, are preferable produced from filamentous fungi because yields are usually higher than those obtained from yeast and bacteria. Indeed, the application of mixed cultures of filamentous fungi (i.e. *Trichoderma reesei* and *Aspergillus niger*) resulted in a positive synergistic effect to obtain higher enzyme production (i.e. xylanase, endoglucanase, amylase, inulinase, β-glucosidases) [5–8]. Numerous enzymes including triacylglycerol lipase (from *Fusarium oxysporum*), trehalase (from *T. reesei*), pectin lyase (from *A. niger*), α-glucosidase (*A. niger*), xylanase (from *Talaromyces leycettanus*), among others, are currently produced by recombinant DNA in *T. reesei* at industrial scale by large companies such as Danisco US Inc, AB Enzymes GmbH, Novozymes, and DuPont [9]. The availability of molecular elements (i.e. promoters, terminators, enhancers) to manipulate gene expression in *Trichoderma* is therefore essential for the successful production of recombinant proteins.

A number of inducible promoter sequences (i.e. *cel7a*, cellobiohydrolase CBH1; *cel5a*, endoglucanase EG2; *xyn1*, xylanase XYN1) derived from xylanase or cellulase genes are available to express homologous and heterologous genes in different *Trichoderma* species [10–12]. The inducible promoter from the *cel7a* gene is one of the most often used for industrial purposes [11, 13]. However, to activate the expression driven by the *cel7a* promoter, high concentrations of an inducer (cellulose, sophorose, and lactose) are required, which can also activate the expression of other genes leading to the production of contaminant proteins. Promoters driving constitutive gene expression are desirable because they allow the production of the protein of interest using simple culture media with no need of an inducer compound or an environmental signal that are rarely specific for a single gene. Moreover, in contrast to the *cel7a* promoter, they are highly active in media containing glucose to repress the expression of cellulose-related and proteases genes reducing downstream processing to produce purer recombinant proteins [14–16]. Although several constitutive promoters useful for the expression of homologous and heterologous proteins in *Trichoderma* have been reported, such as the *rp2* (ribosomal protein), *cDNA1* (unknown), *tef1* (translation elongation factor), *eno1* (enolase), *gpd1* (glyceraldehyde 3-phosphate dehydrogenase), *pdc* (pyruvate decarboxylase), *pki1* (protein kinase) promoters, only some of them are routinely used [15, 17, 18]. A good example is the *pki1* promoter, which is often used to express homologous and heterologous proteins and selectable marker genes (i.e. *hph*) in different *Trichoderma* species [19–23].

In general, approaches to identify potentially useful promoters involve the search for genes highly expressed on cDNA libraries prepared under high glucose conditions or the manual search for orthologues of genes previously reported to be constitutive in other fungal species [15, 17, 24]. More recently, the use of an increasing amount of genomic and transcriptomic data, such as RNA-seq data from different organisms grown under diverse environmental conditions or harvested at different developmental stages has facilitated the identification of promoters with different characteristics, for example, to express genes during specific developmental stages [25].

Most of the evaluations of promoter activity in *Trichoderma* have been done by assessing the expression of genes encoding for xylanases, laccases, and glucoamylases or of reporter genes such as *gfp* and mCherry [12, 17, 18, 20]. In the case of hydrolytic enzymes, it requires to set up laborious protocols to extract the enzymes and assess their functionality under optimal conditions, whereas in the case of fluorescent proteins the constant use of fluorescence microscopy or fluorometers is required to evaluate promoter activity at specific stage of growth. These methods are time consuming and do not allow the direct comparison of different promoters in real time and during the entire growth cycle. Therefore, it would be interesting to develop new tools and approaches to assesses the transcriptional activity of promoter sequences in real time during the entire growth cycle to determine potential detrimental effects on cell growth. To determine if a promoter sequence is functional as a constitutive promoter, we believe that using a dominant and easily detectable growth marker, that allows the continuous supply of an essential nutrient, would bring substantial information in a simpler manner and in a shorter period of time, with no need of enzymatic assays or fluorescence methods.

Here we report P*ccg6*, a new constitutive promoter identified by analysis of RNA-seq data from *T. atroviride* grown under diverse growth conditions. The promoter sequence of *ccg6* orthologues genes displayed conserved arrangements of transcription factor binding sequences among different *Trichoderma* species, potentially involved in their regulatory properties. Transcriptional activity of P*ccg6* was assessed in *T. atroviride* by expressing a codon optimized phosphite oxidoreductase (PTXD) from *Pseudomonas stutzeri* WM88 that allows the conversion of phosphite (Phi), a non-metabolizable-reduced phosphorus (P) chemical form, into phosphate (Pi), an essential nutrient for fungal growth. P*ccg6* was found to drive constitutive expression of the *ptxD* gene to a similar level to that directed by the *pki1* promoter. Transgenic *T. atroviride* lines expressing *ptxD* were able of growing using Phi as the sole source of P in a modified Vogel’s minimal media. Additionally, we propose that a system that allows the selective nutrition of the strain of interest could be used to prevent or reduce biological contamination during *Trichoderma* cultivation for the production of industrial food enzymes.

## Materials and methods

### Fungal strains and routine maintenance

*Trichoderma atroviride* IMI 206040 (TaWT), *Trichoderma reesei* QM6a (Tr) and *Trichoderma virens* Gv29-8 (Tv) were used as *Trichoderma* wild type strains to study the effect of Phi in this work. *Rhizoctonia solani* AG5 was used as the phytopathogenic fungi for antibiosis and confrontations experiments. All the strains were maintained and propagated using potato dextrose agar (PDA, DIFCO™, USA) at 28° C. All the strains were kindly provided by Dr. Alfredo Herrera-Estrella (Centro de Investigacion y de Estudios Avanzados del IPN, in Mexico).

### Plasmid construction

To express the *ptxD* gene in *T. atroviride,* two constructs were generated using the plasmid pCB1004 as backbone [19], which contained an independent hygromycin B resistance-cassette. The coding region of the *ptxD* gene from *Pseudomonas stutzeri* WM88 was used as reference to design and synthesize a DNA sequence (GenBank accession number MN434083) optimized according to the nuclear codon usage of *T. atroviride* using the GenScript’s OptimumGene™ platform. In the first construct, the *ptxD* coding sequence, linked to the 5′UTR and 3′UTR of the *cel7a* (*cbh1*) gene of *T. reesei* [19], was placed under control of the *ccg6* promoter from *T. atroviride* (GenBank accession number MK887357) and the *blu17* terminator. The same arrangement was used in the second construct, except that the *ccg6* promoter was replaced by the *pki1* promoter. The constructs were cloned between the *Kpn*I and *Xba*I sites in the multiple cloning site of pCB1004.

### Genetic transformation

Genetic transformation of *T. atroviride* was carried out according to Herrera-Estrella [26]. Briefly, transformed cells were spread onto PDA plates containing 100 μg mL^−1^ hygromycin B as selective agent. In the darkness *Trichoderma* grows indefinitely as mycelium, whereas light triggers conidiation. Therefore, to favor the production of spores from the transformed cells, plates were incubated in the presence of light until transformants appeared. At least 50 transformants were picked out for each construct, and 15 of them were subjected to five rounds of single spore isolation until stable lines were obtained. Some stable lines were further characterized.

### Transcriptomic data analysis

To analyze the expression pattern of *ccg6* gene, *T. atrovirid*e RNA-seq data were downloaded from the European Nucleotide Archive (https://www.ebi.ac.uk/ena/) and non-wild-type samples were discarded. The following RNA-seq datasets were used for this purpose: (1) PRJNA575031: SRR10207228, SRR10207229, SRR10207220, SRR10207224, SRR10207225; (2) PRJNA310123: SRR2231021, SRR2239861, SRR2239862, SRR2230025, SRR2239855, SRR2239865, SRR2239856, SRR2239866, SRR2239857, SRR2239867, SRR2239868, SRR2239859, SRR2226780, SRR2226739, SRR2239800, SRR2239801, SRR2239792, SRR2239802, SRR2239803, SRR2239794, SRR2239804, SRR2239795, SRR2239796, SRR2239798, SRR2239799 [27]; (3) PRJNA336223: SRR4445669, SRR4445663, SRR4445668, SRR4445662, SRR4445664, SRR4445670, SRR4445667, SRR4445673, SRR4445666, SRR4445672, SRR4445665, SRR4445671; (4) PRJNA476116: SRR7343320, SRR7343321, SRR7343322, SRR7343323, SRR7343324, SRR7343325, SRR7343338, SRR7343339, SRR7343340, SRR7343341, SRR7343342, SRR7343343 [28]; (5) PRJNA508370: SRR8280322, SRR8280323, SRR8280324, SRR8280325, SRR8280326, SRR8280327, SRR8280334, SRR8280335, SRR8280336, SRR8280337, SRR8280338, SRR8280339, SRR8280346, SRR8280347, SRR8280348. A *T. atroviride* transcripts file was created with gffread (http://ccb.jhu.edu/software/stringtie/gff.shtml) using as inputs the *T. atroviride* IMI 206040 (GCA_000171015) TRIAT_v2.0 genome fasta and gff3 files available at EnsemblFungi, and fastq files were aligned and quantified using *kallisto* (version 0.46.1) [29]. Expression data was gene level-summarized using *tximport* [30], and estimated counts were normalized using the GeTMM (Gene length corrected Trimmed Mean of M-values) method [31] to create the corresponding gene expression barplots. GeTMM is a normalization method combining gene-length correction with the normalization procedure TMM [31].

### Analysis of the *ccg6* promoter and CCG6 protein

Multiple alignments of protein sequences were performed using MAFFT (version 7) [32] and the results visualized using the software BoxShade [33]. Phylogenetic tree was generated using the neighbor-joining method and 100 bootstrap resampling using Phylo.io [34]. NCBI accession numbers used in the analyses are as follows: *Trichoderma atroviride* IMI 206040 (*T. atroviride*), XP_013943686.1; *Trichoderma gamsii* (*T. gamsii*), XP_018661560.1; *Trichoderma asperellum* CBS 43.97 (*T. asperellum*), XP_024758524.1; *Trichoderma arundinaceum* (*T. arundinaceum*), RFU72315.1; *Trichoderma harzianum* CBS 226.95 (*T. harzianum*), XP_024772586.1; *Trichoderma virens* Gv29-8 (*T. virens*), XP_013960948.1; *Trichoderma reesei* QM6a (*T. reesei*); XP_006961490.1; *Trichoderma citrinoviride* (*T. citrinoviride*); XP_024751409.1; *Hirsutella minnesotensis 3608* (*H. minnesotensis*), KJ271071.1; *Tolypocladium ophioglossoides* CBS 100239 (*T. ophioglossoides*), POR39196.1; *Neurospora crassa* OR74A (*N. crassa*), XP_960686.2; *Fusarium avenaceum* (*F. avenaceum*), KIL96403.1; and *Fusarium fujikuroi* IMI 58289 (*F. fujikuroi*), XP_023430618.1. Amino acid sequences of the CCG6 proteins from different *Trichoderma* species were analyzed using InterProScan [35] and SignalP version 5.0 servers [36]. Analyses of the *ccg6* promoter sequences of *T. atroviride*, *T. reesei*, *and T. asperellum* to identify DNA motifs were performed using the MEME Suite searching for motifs with minimum and maximum width site of 6 and 50 nucleotides, respectively [37]. Specific DNA motifs were then analyzed in Tomtom [38] using YEASTRACT database to identify matches with reported motifs.

### Molecular analysis

#### Analysis of the presence of the *ptxD* gene by PCR

Genomic DNA was isolated from mycelial tissue using an urea-based method. Briefly, mycelial tissue was harvested, frozen and ground with liquid nitrogen and 600 uL of urea buffer [7 M urea (Sigma-Aldrich, Saint Louis, MO 63103, USA), 0.3 M NaCl (Sigma-Aldrich, Saint Louis, MO 63103, USA), 0.02 M EDTA (Sigma-Aldrich, Saint Louis, MO 63103, USA), 0.05 M Tris–HCl pH 8 (Sigma-Aldrich, Saint Louis, MO 63103, USA)] were added to 100-150 mg of ground tissue, mixed toughly and incubated at room temperature for 30 min. Samples were centrifuged at 10,000 rpm and the supernatant mixed with one volume of isopropanol. Samples were mixed by inversion, incubated for 5–10 min at room temperature, and then centrifuged for 5 min at 10,000 rpm. The pellet was washed using ethanol 70% and DNA resuspended in sterile deionized water. Genomic DNA was quantified in a NanoDrop 2000 spectrophotometer (Thermo Scientific, USA) and its integrity was verified through agarose gel electrophoresis (1%). Possible RNA contamination from DNA samples was removed with RNase A (Invitrogen™, PureLink™, RNase A, Van Alley Way Carlsbad, CA, USA) following manufacturer’s instructions. Later, 500 ng of genomic DNA were used as template to amplify the *ptxD* gene using Taq DNA Polymerase (Invitrogen™, Van Alley Way Carlsbad, CA, USA) according to manufacturer’s instructions. The following conditions were used to amplify the *ptxD* gene: 95 °C for 3 min, 95 °C for 30 s, 65 °C for 30 s, 72 °C for 1 min, 72 °C for 5 min and finally held at 4 °C. Primers used for *ptxD* amplification were *TaptxD*F 5′ ATGCTGCCTAAGCTTGTC 3′ and *TaptxD*R 5′ TCAGCAGGCGGCAGGCTC 3′. PCR product size (840 bp) was verified in agarose gel electrophoresis (1%) using SYBR-Safe DNA Gel Stain (Invitrogen™, Van Alley Way Carlsbad, CA, USA) in order to determine the presence of the transgene.

#### qRT-PCR analysis

To analyze expression levels of the *ptxD* constructs, TaWT and transgenic strains were cultured on PDA plates overlaid with a sterile cellophane membrane and incubated for 3 days at 28 °C. Then, mycelia were harvested, frozen and ground with liquid nitrogen. Total RNA isolation was carried out using PureZOL™ (BIORAD, Hercules, CA, USA) isolation reagent, according to manufacturer’s instructions. Total RNA concentration was determined using a NanoDrop 2000 spectrophotometer (Thermo Scientific, USA) and its integrity and quality was verified through agarose gel electrophoresis (1.5%). Afterwards, 1 µg of total RNA was treated with DNase I (Invitrogen™, Van Alley Way Carlsbad, CA, USA) to remove any possible genomic DNA and cleaned up using the RNeasy^®^ Mini Kit (Qiagen) according manufacturer’s instructions. Briefly, total RNA was reversed-transcribed to cDNA with SuperScript III Reverse Trancriptase (Invitrogen™, Van Alley Way Carlsbad, CA, USA), according to manufacturer’s instructions. Oligonucleotides used for *ptxD* amplification were: Ta*ptxD*F 5′-ATGCTGCCTAAGCTTGTC-3′ and Ta*ptxD*R 5′-TCAGCAGGCGGCAGGCTC-3′. Real Time qPCR was performed in quadruplicates for each sample using a 7500 Real Time System and the SYBR^®^ Green Master Mix (Applied Biosystems, USA), according to the manufacturer’s instructions. *T. atroviride* glyceraldehyde-3-phosphate dehydrogenase (*gpd,* Id 297741) gene was used as reference and was amplified in parallel with the *ptxD* gene. Primers used to amplify the *gpd* gene were *gpd*F 5′-GTGCTGCCCAGAACATCATCC-3′ and *gpd*R 5′-TGGCGGTAGGGACACGAATG-3′. Standard curves were obtained using five-serial-dilutions for *ptxD* and *gpd* genes. The data were analyzed with the 2^−ΔΔCt^ method to determine the expression of the *ptxD* gene.

To analyze the expression levels of the *ccg6* gene, TaWT was cultured for 4 days at 28 °C in darkness on liquid VMM supplemented with 2% glucose, sucrose and mannitol as the carbon source, and under carbon (0.26%) and nitrogen (0.003 mM) starvation, and in PDB as control. Additionally, TaWT was cultured on both liquid PDB and VMM at 28 °C in darkness, and mycelium collected 2, 4 and 6 days after inoculation. Mycelia were harvested, frozen and ground with liquid nitrogen and total RNA was isolated using PureZOL™ (BIORAD, Hercules, CA, USA) and processed as described above. Real Time qPCR was performed in triplicates for each sample using the Q-qPCR instrument (Quantabio, Germany) and the PerfeCTa^®^ SYBR^®^ Green FastMix^®^ (Quantabio, Germany), according to the manufacturer’s instructions. *T. atroviride gpd* (Id 297741), and *elF*-*4* (Id 301614) genes were used as qRT-PCR expression controls. Primers used to amplify *ccg6, gpd*, and *elF*-*4* control genes were: *ccg6*F5′-CGACACACCTCGCCAATATAC-3′, *ccg6*R5′-GTAGCGCATCTTCTCGTG-3′; *gpd*F5′-GCTGCCGATGGTGAGCTCAAGGG-3′ and *gpd*R5′-GAGGTCGAGGACACGGCGGGA-3′, and *elF*-*4*F5′-GTCCAACTACGATGAGACTGTC-3′ and *elF*-*4*F 5′-TCGTGGCCCTTGATAACAG-3′, respectively. The data were analyzed with the 2^−ΔΔCt^ method to determine the expression levels of the *ccg6* gene.

#### PTXD enzymatic activity

The enzymatic activity of PTXD was determined using total protein cell extracts. Flasks with 50 mL of Vogel’s Minimal medium (VMM) [39] supplemented with 36 mM Pi (KH_2_PO_4_) for WT strain and 1 mM Phi (KH_2_PO_3_) for transgenic strains, were inoculated with 1x10^7^ conidia and cultured at 200 rpm, 28 °C for 7 days. Mycelial growth was harvested and resuspended in 5 mL of resuspension buffer [50 mM MOPS (Sigma-Aldrich, Saint Louis, MO 63103, USA) pH 7.5, 25 mM NaCl (Sigma-Aldrich, Saint Louis, MO 63103, USA), 1 mM EDTA (Sigma-Aldrich, Saint Louis, MO 63103, USA), and 50 µL of Protease Inhibitor Cocktail (Sigma-Aldrich, Saint Louis, MO 63103, USA)] and cells were lysed by ultrasonication on ice using an Ultrasonic Processor (Cole-Parmer, Vernon Hills, IL, USA) with 40% amplitude, 2 cycles of 4 min with short bursts of 10 s followed by intervals of 5 s for cooling on ice. Then, samples were centrifuged at 9000 rpm at 4 °C for 20 min. The resulting supernatant was applied to a 10 K MWCO dialysis tubing (SnakeSkin Dialysis Tubing, ThermoFisher Scientific, Rockford, IL, USA) and incubated at 4 °C for 24 h. Samples were quantified using Quick Start™ Bradford Dye Reagent (BIORAD, Hercules, CA, USA) according to manufacturer’s instructions in a 96 well clear microtiter plate (Corning Inc, USA). Extracts were adjusted to the same protein concentration (0.5 mg mL^−1^). Determination of PTXD enzymatic activity was performed using a fluorescence-based method for NADH detection. Each reaction was started in individual wells of a 96 well black microtiter plate by adding 100 µL assay mix to give a final concentration of 50 mM MOPS, 0.5 mM Na_3_PO_3_ (Sigma-Aldrich, Saint Louis, MO 63103, USA) pH 7.0, 0.75 mM NAD^+^ (Sigma-Aldrich, Saint Louis, MO 63103, USA), and 25 µg µL^−1^ of protein extract per well. The microtiter plate was incubated at 37 °C for 1 h in the darkness. The reaction product was quantified with a fluorescence reader Fluoroskan Ascent™ FL (Thermo Fisher Scientific, Vantaa, Finland) at 340 nm excitation and 460 nm emission wavelengths for NADH detection.

### Phenotypic characterization

#### Antagonism assays

TaWT and transgenic strains were cultured on PDA plates overlaid with a sterile cellophane sheet, inoculated with 1 × 10^5^ conidia suspension, and incubated at 28 °C for 48 h. The cellophane sheet together with the fungal colony was removed from cultures; plates were then inoculated with *R. solani* AG5 mycelial plugs. The plates were incubated at 28 °C for 72 h in total darkness and then photographed to determine fungal colony growth.

#### Mycoparasitism assays

Mycoparasitism of TaWT and transgenic strains was evaluated against *R. solani* AG5. Mycelial plugs from *R. solani* AG5, taken from the edge of actively growing colonies of fresh fungal cultures and 1x10^5^ conidia suspension from TaWT and transgenic strains were cultured on PDA, placed approximately 5 cm from each other. Plates were incubated at 28 °C for 96 h in total darkness and then photographed to determine fungal colony growth.

### Evaluation of the capacity of *Trichoderma* wild type strains to metabolize phosphite

To evaluate the capacity of TaWT, Tr, and Tv of growing using Phi as the P source, we used a modified VMM recipe, in which the source of P, monopotassium phosphate, was replaced by monopotassium phosphite. VMM is commonly used to cultivate *Neurospora crassa*, however, it has been also successfully used to grow *Trichoderma* species including TaWT, Tr, Tv, and *T. asperellum* [40–43]. VMM was prepared as a 50× solution and used as a 1× solution and supplemented with glucose 2% and Bacto Agar (DIFCO™, USA) 1.5% when needed. The P sources were monopotassium phosphate (Pi, KH_2_PO_4_, Sigma-Aldrich, USA) or monopotassium phosphite (Phi, KH_2_PO_3_, Wanjie International Co., Limited, China), as established for each experiment. A 1 × 10^5^ conidia suspension from TaWT was inoculated in Petri dishes containing VMM supplemented with 36 mM Pi and incubated at 28 °C for 36 h. For experiments in which mycelium plugs were used as inoculum, mycelial plugs taken from the actively growing colony were placed in new Petri dishes with PDA, VMM without a P source and VMM supplemented with Pi (36 mM) or Phi (1, 2, 3, 4, or 5 mM) at the P sources. Plates were incubated at 28 °C for 8 days in total darkness, and then colony growth was evaluated. Similar experiments were performed but using TaWT conidia (1 × 10^5^) as inoculum in solid VMM media. Additionally, experiments in glass flaks containing liquid VMM media without a P source, supplemented with 36 mM Pi, and 1, 2, 4, and 5 mM Phi, and inoculated with 1 × 10^5^ TaWT conidia suspension were also performed (28 °C, 200 rpm, 8 days, constant light). TaWT conidia were obtained from PDA cultures by exposing them to constant white light during 96 h. Conidia were collected by scraping the culture surface with sterile water and counted in a Neubauer chamber.

Experiments to evaluate the capacity of Tr and Tv to metabolize Phi, were performed as mentioned before in VMM solid media, but both mycelium plugs and conidia inoculum were produced from fresh cultures grown on VMM with Pi (10 mM). Inoculum were placed in Petri dishes containing PDA, VMM media without a P source, supplemented with 36 mM Pi, and 0.25, 0.5, 0.75, 1, and 2 mM Phi. Colony growth was determined by measuring colony area (cm^2^) using ImageJ 2.0.0-rc-43/1.50e [44].

### Growth of transgenic strains using phosphite as the sole phosphorus source

To determine the ability of transgenic strains to grow using Phi as sole P source, transgenic and TaWT strains were cultured in flasks containing 30 mL of VMM supplemented with 1, 2, 4, and 5 mM Phi. Each glass flask was inoculated with 1 × 10^7^ conidia suspension from each *Trichoderma* strain. VMM media with Pi (36 mM) and without a P source were used as controls. Flasks were incubated at 28 °C and 200 rpm for 7 days. Afterwards, biomass produced for each *Trichoderma* strain (dry weight, DW) was determined by filtration of the culture with a sterile Whatman filter paper and dry weight determined.

### Competition experiments

Competition experiments were performed at 28 °C and 200 rpm in 50 mL glass flasks containing 25 mL of VMM without a P source, supplemented with 10 mM Pi or 4 mM Phi as P source. 1 × 10^8^ conidia of transgenic strain *ccg6*_OPT_-3 were co-cultivated with 50 μL inoculum of a culture of *E. coli* DH5α. For the preparation of the inoculum of *E. coli* DH5α, 50 µL of glycerol stock was activated with 500 μL of SOC medium, incubated at 37 °C and 200 rpm during 2 h. Monocultures were performed in the same conditions. At the end of 1 week of cultivation, mycelia were harvested by filtration with a sterile Whatman filter paper and dry weight determined. CFU were determined in the filtrate.

Adjustments on brightness, sharpness, and contrast were applied to photographs of Petri dishes and glass flasks in Figs. 3, 4, 5, 6, Additional file 1: Figures S3, S4, S5, S6, S8, and S9 to improve image quality and visibility of the mycelium. We did not obscure or eliminate any information present in the originals.

### Nucleotide sequences

Sequences of the *ccg6* promoter and the *ptxD* gene codon-optimized for *T. atroviride* were submitted to the GenBank database under accession numbers MK887357 and MN434083, respectively.

### Statistical analysis

Statistical analyses were performed using a one-way ANOVA and Tukey HSD test (*p* < 0.05).

## Results

### Identification of the *ccg6* promoter in *T. atroviride*

**Fig. 1:**
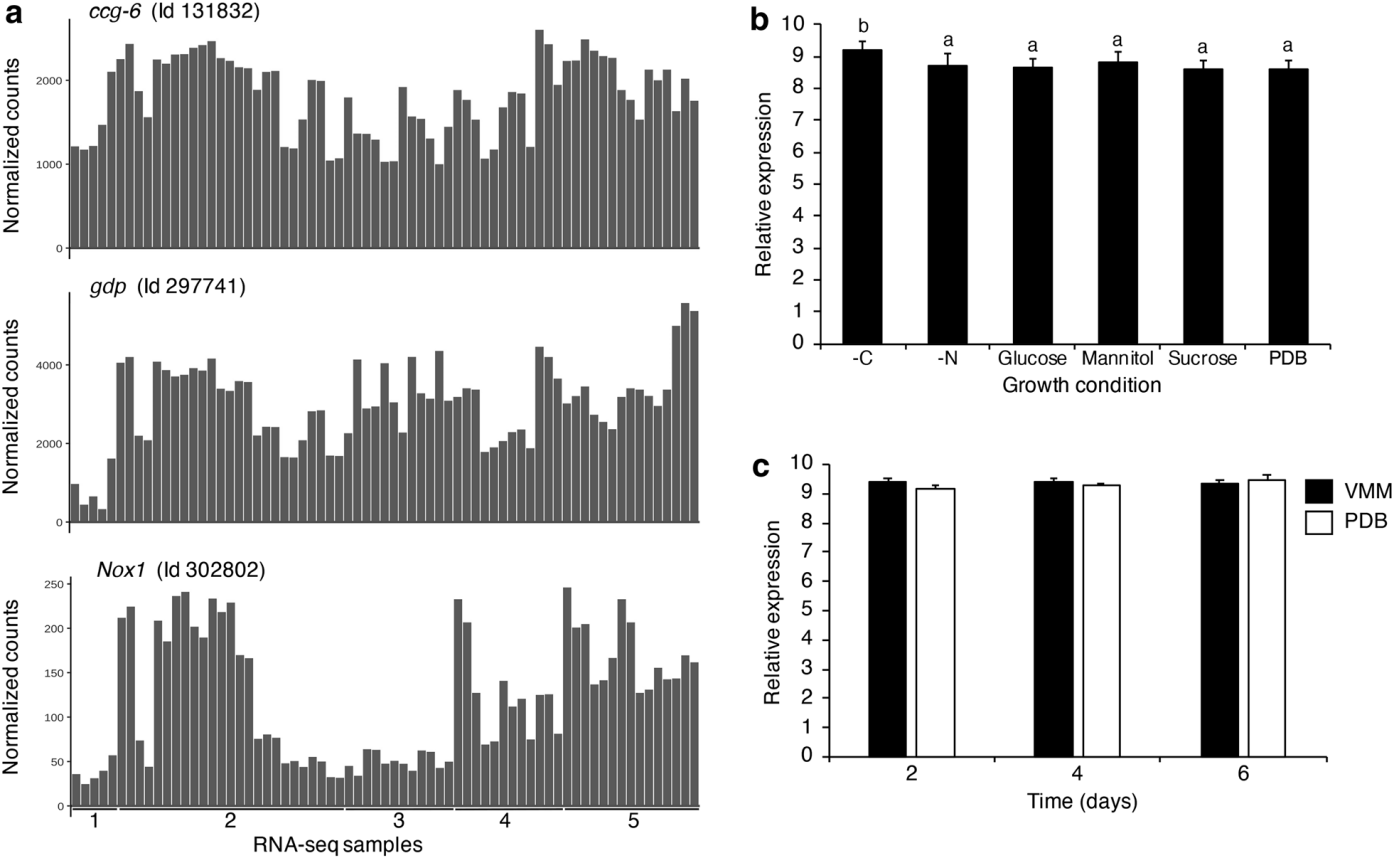
Transcriptional activity of *ccg6* gene in *Trichoderma atroviride*. **a** Transcriptomic profile of *ccg6*, *gdp and Knox1* genes in *Trichoderma atroviride* IMI 206040 (TaWT) according RNA-seq data sets publicly available at the European Nucleotide Archive (https://www.ebi.ac.uk/ena/) and organized into five groups: 1, 2, 3, 4 and 5 (see Materials and Methods section). Expression data was gene level-summarized [30], and estimated counts were normalized using the GeTMM (Gene length corrected Trimmed Mean of M-values) method [31]. **b** qRT-PCR analysis of the *ccg6* gene in *T. atroviride* grown under different conditions including carbon (-C) and nitrogen starvation (-N), and using different carbon sources (2% of glucose, sucrose or mannitol). Culture in potato dextrose broth (PDB) media was used as control treatment. **c** qRT-PCR analysis of the *ccg6* gene in *T. atroviride* grown in Volgel’s minimal media (VMM) and PDB at 2, 4 and 6 days after inoculation. The *gpd* and *elF*-*4* genes were used as internal controls. Data are expressed as mean ± SE, n = 3 (*p *< 0.05). Different letters indicate statistical difference

Environmental stimuli greatly influence the development of filamentous fungi. *In T. atroviride*, light, carbon and nitrogen starvation, and mechanical damage triggers asexual reproduction [27, 45, 46]. Moreover, a large variety of processes including sexual and asexual development, growth rate, as well as cellular metabolism and gene expression, are regulated by the circadian system. In total darkness, *T. atroviride* grows indefinitely as a mycelium, whereas a simple pulse of blue light induces the formation of the so called “conidiation ring” at the edge of the colony [47]. High-throughput RNA-seq analyses have tremendously facilitated the study of the transcriptional responses of *T. atroviride* to variable environmental conditions and allowed the elucidation of genes and mechanisms regulating these processes. RNA-seq analysis provide gene expression profiles at wide scale which can be exploited for different purposes. To identify genes with a consistent or constitutive expression under different conditions, we mined publicly available RNA-seq databases from *T. atroviride* IMI 206040 (TaWT) grown under different conditions such as darkness, blue light, constant white light, and mechanical injury [27, 45, 48, 49], and analyzed the level of gene expression. We found that the transcript of the *Taccg6* gene (Id 131832 in the *T. atroviride* TRIAT_v2.0 genome available at EnsemblFungi) [50], is present at high levels in the different datasets analyzed (Fig. 1a). The analysis also showed that the *ccg6* transcript levels were relatively constant in the different growth conditions for which *T. atroviride* RNA-seq datasets are available. *ccg6* transcript levels were more constant (between 1100 and 2300 GeTMM) (see Materials and Methods section) under different conditions, than those of the *gdp* (Id 297741, which varied between 200 and 5000 GeTMM) a highly expressed gene that is commonly used as a stable internal control for quantitative real time PCR (RT-qPCR) expression analysis (Fig. 1a). *ccg6* transcript levels are substantially higher and much lees variable than those of the *Knox*1 (Id 302802) gene, that is highly responsive to mechanical injury [45]. To validate that *ccg6* displays a constitutive pattern of expression independent of growth conditions, we determined using qRT-PCR the *ccg6* transcript levels in RNA extracted from *T. atroviride* cultivated under distinct growth conditions, such as different carbon sources (glucose, sucrose and mannitol), under carbon and nitrogen starvation and in standard rich medium (Potato Dextrose Broth). We found that *ccg6* had a relatively constant level of expression varying from 8.57 to 9.18 in the relative transcript levels respect to the two genes used as constitutive controls (Fig. 1b). In addition, *ccg6* expression level was analyzed at three different times (days 2, 4, and 6) of *T. atroviride* growth in two different liquid media, PDB and Vogel’s Minimal Media (Fig. 1c). No significant statistical difference in the *ccg6* transcript level were observed at the different time points in either of the two growth media. Together, the RNA-seq data and RT-qPCR showed that the levels of *ccg6* transcript are constant under different conditions, and therefore, suggest *ccg6* promoter sequence behaves as a constitutive promoter in *T. atroviride.*

Alignment of the predicted CCG6 protein of TaWT with orthologue sequences from other fungal species showed high similarities among proteins from different *Trichoderma* species and to a lower extent to that of *N. crassa*, which additionally had an 80 aa N-terminal extension that is missing in the proteins encoded by genes from all other fungal species analyzed (Additional file 1: Figure S1a). The presence of this additional fragment in the *N. crassa* CCG6 protein suggest that it might have a different biological function than those of *Trichoderma* and other fungal species. Phylogenetic analysis showed that the CCG6 proteins from *Trichoderma* species form a discrete clade compared to those of other filamentous fungi (Additional file 1: Figure S1b). CCG6 proteins of *Trichoderma* species were analyzed using InterProScan [35] and SignalP [36] to identify putative conserved domains and signal peptides. The analyses suggest that CCG6 belong to the SED1/SPI1 (Suppression of Exponential Defect1/Stationary Phase Induced1) family of cell wall-anchored proteins displaying a non-cytoplasmic C-terminal domain, which contain a potential signal peptide (MKFTAAVALAAV(A)GVSA) in the N-terminal part of the protein (Additional file 1: Figure S1a). Proteins belonging SED1/SPI1 family have been mainly studied in *S. cerevisiae* and reported to have important functions in cell wall structure and biogenesis, and are induced during stress conditions [51–53]*. Spi1* for example, is induced under a number of stress conditions such as nutrient starvation, hyperosmotic and oxidative stress, among others [52].

**Fig. 2:**
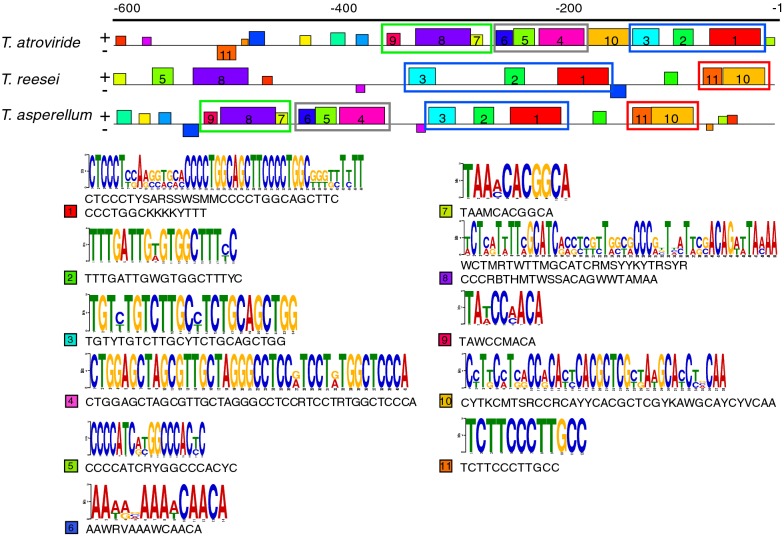
DNA motifs identified in the *ccg6* promoter. Comparative analysis of a 600 bp sequence upstream the transcription start site of *T. atroviride*, *T. reesei,* and *T. asperellum ccg6* gene using the MEME suite. Identified DNA motifs are numbered from 1 to 11, and organized into groups: 1 (blue frame), 2 (gray frame), 3 (green frame), and 4 (orange frame). Logos and consensus sequence for DNA motifs 1 to 11 are shown

We denominated P*ccg6* to the promoter sequence of *Taccg6* (GenBank accession number MK887357) of *T. atroviride* IMI20604. To gain insight about the transcriptional regulation of *ccg6*, we searched for DNA motifs potentially conserved in the *ccg6* promoter region. With this aim a 600 bp region upstream of the transcription start site of *ccg6* genes in *T. atroviride*, *T. reesei*, and *T. asperellum* was analyzed using the MEME suite. We identify common putative DNA motifs distributed along the sequence between the three promoters, some of which were disposed in specific arrangements (Fig. 2). To easily compare DNA motifs present in the promoters, we organized the motifs into four groups; group 1 (motifs 1, 2, 3), group 2 (motif 4, 5, 6), group 3 (motifs 7, 8, 9), and group 4 (motifs 11 and 10) (Fig. 2). We found that groups 1, 2, and 3 are in the same order in the *ccg6* promoter of *T. atroviride* and *T. asperellum* (Fig. 2). In *T. reesei*, we found only group 1, and apparently an inverted version of group 3 present upstream with motif 5 inserted between motifs 7 and 8. In addition, group 4, comprising motifs 10 and 11, was also present in the three promoters, however, motifs 10 and 11 were next to each other in *T. reesei* and *T. asperellum*, immediately upstream of the transcriptional start site, whereas in *T. atroviride* both motifs were located apart of each other (Fig. 2). We then used these DNA motifs to search for similarity against a collection of yeast transcription factor (TF) binding site motifs. We found that some of the motifs identified in P*ccg6* closely resembled the binding motif of different types of TFs including zinc-finger, basic leucine zipper, and Myb-type TFs; among these TFs are Aft1p, Crz1p, Haa1p, Hsf1p, Mig1/2/3p, Hac1p, Rox1p, Ste12p, Ino4p, and Ino2p (Table 1). Northern blot hybridization, expression microarrays and chromatin immunoprecipitation analyses support that most of these TFs regulate the expression of *Spi1* in *S. cerevisiae* [51, 54–56]. *Spi1* harbors potential binding sites for the TFs Ash1p, Crz1p, Cbf1p, Fkh1/2p, Gcr1p, Hac1p, Hsf1p, Nrg1, Ste12p TFs among others, which are potentially involved in its transcriptional regulation [52].

**Table 1:** Transcription factors potentially binding the identified DNA motifs in the *ccg6* promoters analyzed, using the MEME suite

Motif	Transcription factors
1	Haa1p, Gcr1p, Hsf1p, Reb1p
2	Nrg1p, Snk7p, Abf1p
3	Basp1p, Yrm1p, Hac1p, Rfx1p, Pdh1p, Gsm1p
4	Met31p, Met32p
5	Rap1p, Met32p, Met31p, Hcm1p, Ndt80p, Sum1p, Matalpha2p, Rox1p, Crz1p, Rap1p
6	Rfx1p, Met32p, Nrg2p, Cbf1p, Nrg1p, Crz1p
7	Fkh2p, Mig1p, Mig2p, Mig3p, Ume6p, Mbp1p, Ndt80p, Stb5p, Stp1p, Yrr1p, Rfx1p, Sut2p
8	Rfx1p, Ste12p, Swi4p, Met31p, Met32p, Yrr1p, Rfx1p
9	Ino4p, Mot3p, Haa1p, Gcr1p, Ino2p, Mga1p
10	Hac1p, Rsc30p, Arg81p, Cbf1p, Aft2p, Met32p, Aft1p
11	Ash1p, Arg81p, Rap1p

Motifs are numbered according Fig. 1

### Construction of *ccg6*_OPT_*::ptxD* and *pki*_OPT_::*ptxD*

To determine if the *ccg6* sequence promoter is functional as a constitutive promoter, we selected as growth reporter gene the coding sequence of the *ptxD* gene from *Pseudomonas stutzeri* WM88 that encodes a phosphite oxidoreductase (PTXD). PTXD converts Phi into phosphate allowing organisms that express this enzyme to use Phi as a sole P source, a trait that is not present in most eukaryotic and is present only in few bacterial strains [57, 58]. Therefore, PTXD can act as a growth reporter gene, for which enzymatic assays are also available [59, 60]. However, there is no information about the functionality of the system in filamentous fungi. To test whether a 600 bp fragment of the *Taccg6* promoter, containing most of the conserved array of TF binding sites, we constructed chimeric genes fusing the *Taccg6* promoter with the coding sequence of *ptxD* (Additional file 1: Figure S2). To be able to compare expression level driven by the *ccg6* promoter with that of a known promoter, we selected P*pki1*, a widely used constitutive promoter for gene expression in *Trichoderma* species, which was also fused to the *ptxD* coding sequence (Additional file 1: Figure S2). For both constructs a codon-optimized version of the *ptxD* gene for expression in TaWT (submitted to the GenBank database under accession number MN434083) and the pCB1004 vector as backbone were used [19].

### *T. atroviride* is unable to metabolize Phi as sole P source

**Fig. 3:**
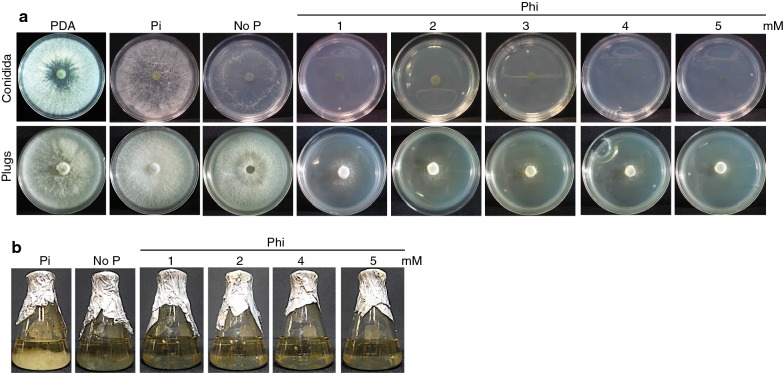
Effect of phosphite in the growth of *T. atroviride*. **a***T. atroviride* IMI 206040 mycelium plugs or conidia (1 × 10^5^) were inoculated in solid modified Vogel’s minimal media supplemented with different concentrations of phosphite (Phi; 1, 2, 3, 4, and 5 mM) as the sole phosphorus (P) source. Media without P (No P), with phosphate (Pi) as the P source and PDA were used as controls. **b***T. atroviride* conidia (1 × 10^5^) were inoculated in liquid modified Vogel’s minimal media supplemented with different concentrations of phosphite (Phi; 1, 2, 4, and 5 mM) as the sole phosphorus (P) source. Media without P (No P) and with phosphate (Pi) as the P source were used as controls. Glass flasks were photographed after 7 days of incubation

In principle, the *ptxD*/Phi system should work for any organism unable to naturally metabolize Phi. To test the potential use of *ptxD* as a growth marker in *T. atroviride,* we first determined whether this fungal species is naturally capable of using Phi as the sole P source. With this aim, we used the Vogel’s minimal media (VMM) devoid of phosphate and supplemented with different concentrations of phosphite (Phi) or phosphate (Pi) to study the growth of a wild-type strain of *T. atroviride* (TaWT). For these experiments we used the two commonly sources of *T. atroviride* inoculum for propagation, mycelium plugs and conidia. TaWT was inoculated in solid VMM containing 1, 2, 3, 4, and 5 mM Phi as sole P source to compare its growth with that displayed in Pi-containing media (Pi) and media lacking P (No P); standard PDA rich medium was used as control. In the case of experiments using TaWT mycelium plugs, we observed a very clear and abundant growth in PDA and Pi-containing media covering an area in the dish of 59.99 and 58.45 cm^2^, respectively, with no statistical difference in colony area. In media lacking P, we observed abundant growth, but slightly decreased colony area (52.88 cm^2^) as compared to PDA and Pi controls (Fig. 3a, Table 2). TaWT growth in media lacking Pi is probably due to the presence of high amount of Pi in the agar plug itself used in the inoculum and/or to high reserves accumulated in the fungus during the initial propagation as the standard VMM medium contains 36 mM Pi. Interestingly, when the mycelium plugs were inoculated in media containing Phi, we observed that colony development was severely inhibited when compared to the growth observed in Pi, No P, and PDA media (Fig. 3a, Table 2). Inhibition of TaWT growth was Phi concentration-dependent, as determined by the area of the colony, ranging from 12.21 cm^2^ in 1 mM Phi to less than 3 cm^2^ in 5 mM Phi, including the area of the plug (~ 2 cm^2^). Although non-toxic effects have been reported for Phi, growth inhibition has also been reported in plants and microalgae probably due to a competition with Pi for transporters to entry into the cell or by inhibition of enzymatic reactions that require Pi. To test whether the presence of Pi in the agar plug or Pi accumulated in the mycelia was responsible for the observed growth in media lacking Pi or supplemented with Phi, we inoculated fresh plates with mycelium plug produced from VMM plates without a P source (such as that shown in Fig. 3a). When the Pi-depleted mycelia was transferred to 2 mM Phi, the growth of TaWT was completely inhibited, but when the inoculum was produced in media containing 2.5 mM Pi a certain degree of growth was observed (Additional file 1: Figure S3), confirming that either the agar plug or Pi accumulated in the mycelia allowed growth in the presence of Phi.

**Table 2:** Colony area (cm^2^) of *T. atroviride* grown in VMM media supplemented with different concentrations of phosphite (Phi; 1, 2, 3, 4, and 5 mM)

	PDA	No P	Pi	Phi				
				1	2	3	4	5
Conidia	60.6 ± 0.36	36.7 ± 0.39*****	50 ± 0.12****	0.6 ± 0.12	0.7 ± 0.05	0.7 ± 0.11	0.7 ± 0.08	0.6 ± 0.12
Mycelium plugs	59.9 ± 0.05	52.8 ± 0.56*****	58.4 ± 0.46	12.2 ± 0.12*****	8.3 ± 0.24*****	7.0 ± 0.48*****	5.9 ± 0.92*****	2.9 ± 0.55*****

Vogel’s minimal media (VMM) with phosphate (Pi) and without phosphorus (P) source (No P), and PDA used as control treatments. Conidia and mycelium plugs were used as inoculum. The values are indicated as the mean value ± SE (n = 3)

Colony area included the spot where the inoculum was placed

***** Significant at *p *< 0.00001; ANOVA-Tukey HSD

When the experiments were carried out using conidia as inoculum, we observed that TaWT grew vigorously in PDA media and displayed similar colony area as when mycelia plugs were used as inoculum (Fig. 3a, Table 2). However, in VMM containing Pi, TaWT formed colonies with a less dense mycelial mat and about 14% less area than those observed when mycelium plugs were used as inoculum. In media lacking Pi, TaWT conidia was also able to grow but with a much less dense mycelial mat with about 27% smaller colony area than that observed in Pi containing media (Fig. 3a, Table 2). In media containing Phi as a sole P source, no growth was detected at any concentration, and in fact growth inhibition was observed in the central part of the dish were the drop containing conidia was deposited (Fig. 3a, Table 2). Similar results were observed by germinating conidia in liquid MVV supplemented with the same Phi concentrations (Fig. 3b). These results demonstrate that *T. atroviride* IMI 206040 is unable to use Phi as a P source, which instead exerts an inhibitory effect on its growth.

**Fig. 4:**
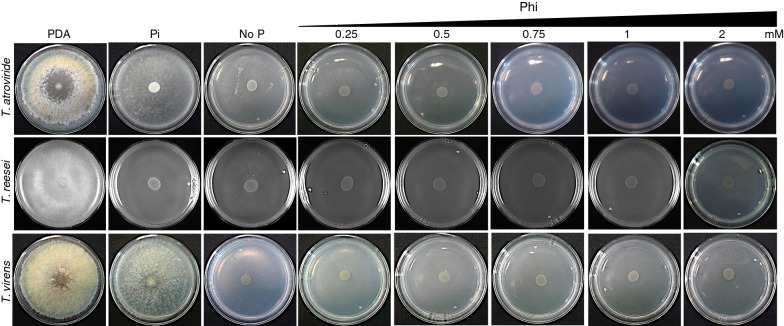
Effect of phosphite in the growth of *T. virens* and *T reesei*. *T. atroviride* IMI 206040, *T. reesei* QM6a, and *T. virens* Gv29-8 conidia (1 × 10^5^) were inoculated in modified Vogel’s minimal media supplemented with different concentrations of phosphite (Phi; 0.25, 0.5, 0.75, 1, and 2 mM) as the sole phosphorus (P) source. Media without P (No P), with phosphate (Pi) as the P source, and PDA were used as controls

To test whether other *Trichoderma* species are unable to use Phi as a P source, we tested the growth of Tr and *T. virens* Gv29-8 (Tv) in VMM supplemented with Phi concentrations (0.25, 0.5, 0.75, 1 and 2 mM) and including Pi, No P, and PDA media as controls. TaWT was also cultured in the same media. The three *Trichoderma* species displayed abundant growth in PDA media showing that the conidia inoculum was viable (Fig. 4, Table 3). When the three *Trichoderma* species were cultured in VMM containing Pi as P source, we observed that TaWT and Tv covered most of the Petri dish with a dense mycelial mat, whereas Tr covered a smaller are of the Petri dish with less dense growth. The observed reduced growth of Tr is probably because VMM is not the optimal growth media for this *Trichoderma* species (Fig. 4, Table 3). When cultured in media lacking Pi, the three *Trichoderma* species displayed a less dense growth than that observed under Pi media and a decreased colony area of 41, 32 and 26% for TaWT, Tr and Tv, respectively, as compared to their growth in Pi media (Fig. 4, Table 3). When conidia were inoculated in Phi-containing media, the growth of the three *Trichoderma* species was completely inhibited in media containing 1 and 2 mM Phi (Fig. 4, Table 3). However, TaWT and Tv displayed some degree of visible growth in 0.25 and 0.5 mM Phi (Fig. 4, Table 3). A similar behavior was observed for the three *Trichoderma* species when mycelium plug was used as inoculum (Additional file 1: Figure S4, Table 3). These results show that all *Trichoderma* species have Pi reserves or are able to scavenge traces of Pi presents as contaminant in the media to allow some degree of growth in media devoid of Pi and, more importantly, that Tv and Tr are also unable to metabolize Phi as P source.

**Table 3:** Colony area (cm^2^) of *T. atroviride*, *T. reesei*, and *T. virens* grown in VMM supplemented with different concentrations of phosphite (Phi; 0.25, 0.5, 0.75, 1, and 2 mM)

	*T. atroviride*	*T. reesei*	*T. virens*
*Conidia*			
PDA	58.1 ± 0.2***	60.1 ± 0.5	58.1 ± 0.4
No P	28.5 ± 0.1*****	29.6 ± 0.3*****	36.9 ± 0.8*****
Pi	49.0 ± 0.1***	43.7 ± 0.5*****	50.5 ± 0.5
0.25 Phi	17.3 ± 0.5*****	1.6 ± 0.4*****	32.1 ± 0.8****
0.5 Phi	5.1 ± 0.2*****	1.3 ± 0.2*****	11.3 ± 0.4*****
0.75 Phi	2.0 ± 0.1*****	1.1 ± 0.04*****	4.6 ± 0.3*****
1 Phi	1.1 ± 0.07*****	1.0 ± 0.004*****	1.2 ± 0.02*****
2 Phi	0.9 ± 0.0*****	1.0 ± 0.04*****	1.0 ± 0.03*****
*Mycelium plugs*			
PDA	53.7 ± 0.5*****	59.8 ± 0.3	53.8 ± 0.6*****
No P	38.3 ± 0.3*****	24.1 ± 0.5*****	44.8 ± 0.6*****
Pi	55.9 ± 0.9*****	42.1 ± *****	51.6 ± 0.5
0.25 Phi	34.7 ± 0.6**	31.4 ± 0.4*****	38.6 ± 0.2*****
0.5 Phi	20.0 ± 0.8*****	29.9 ± 0.6*****	31.2 ± 0.2*****
0.75 Phi	17.7 ± 0.5*****	23.6 ± 0.4*****	22.3 ± 0.3**
1 Phi	11.8 ± 0.6*****	22.3 ± 0.4*****	18.4 ± 0.3*****
2 Phi	4.2 ± 0.2*****	15.1 ± 0.2*****	12.1 ± 0.1*****

Vogel’s minimal media (VMM) with phosphate (Pi) and without phosphorus (P) source (No P), and potato dextrose agar (PDA) used as control treatments. Conidia and mycelium plugs were used as inoculum. The values are indicated as the mean value ± SE (n = 3)

Colony area included the spot where the inoculum was placed

** Significant at *p *< 0.01, *** significant at *p *< 0.001, ***** significant at *p *< 0.00001; ANOVA-Tukey HSD

### Phosphite metabolism can be used as a dominant growth marker in *T. atroviride*

To investigate whether the *ccg6* promoter can be used to express heterologous genes in *T. atroviride*, vectors *pki1*_OPT_ and the *ccg6*_OPT_ were transformed into TaWT via a previously reported protocol [26]. The hygromycin B resistance cassette present in pCB1004, in which the hygromycin B phosphotransferase gene is under control of the Amp (*bla*) promoter, was used as a selectable marker for the transformation process. After protoplast transformation, at least fifty primary hygromycin resistant colonies were obtained for each construct. Fifteen of these hygromycin resistant colonies were subjected to five rounds of single spore isolation until stable lines were obtained. The transformants obtained were picked out onto PDA plates containing hygromycin (100 μg mL^−1^) for routine conservation.

**Fig. 5:**
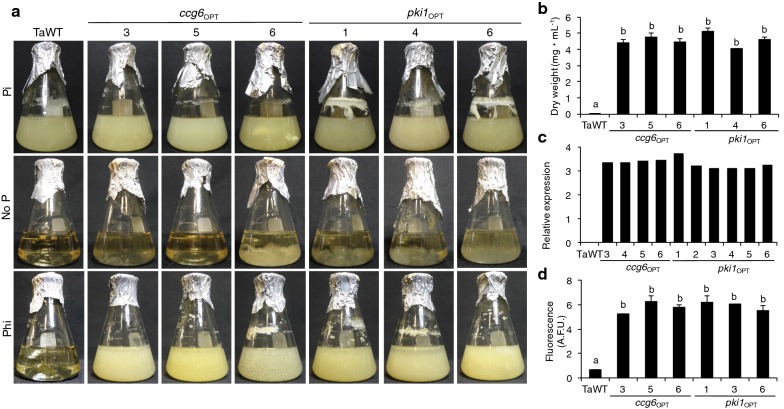
Characterization and growth of *T. atroviride* transgenic strains in media supplemented with phosphite. **a** Growth of six *Trichoderma* transformants (*ccg6*_OPT_-3, -5, -6 and *pki1*_OPT_-1, -4, -6) harboring *ccg6*_OPT_ and *pki1*_OPT_ constructs and the *Trichoderma* wild type strain (TaWT) in modified liquid Vogel’s minimal media supplemented with 1 mM phosphite (Phi) as the phosphorus (P) source. Media without P (No P) and with phosphate (Pi) as the P source were used as controls. Cultures were photographed 7 days after the inoculation. **b** Dry weight (mg mL^−1^) of the mycelia produced by TaWT and transgenic strains in (**a**). Data are expressed as mean ± SE, n = 3 (*p *< 0.05). Different letters indicate statistical difference. **c** Relative expression of the *ptxD* gene in *Trichoderma* transgenic strains *ccg6*_OPT_-3, -4, -5, and -6, and *pki1*_OPT_-1, -2, -3, -4, -5, -6 determined by quantitative real time PCR (qRT-PCR). *T. atroviride* IMI 206040 (TaWT) strain and *gpd* (glyceraldehyde-3-phosphate dehydrogenase) gene were used as negative and internal controls, respectively. **d** PTXD enzymatic activity expressed as Absolute Fluorometric Units (A.F.U.) determined in *T. atroviride* transgenic strains *ccg6*_OPT_-3, -5, and -6, and *pki1*_OPT_-1, -3, and -6, and TaWT as negative control

Initial experiments using modified VMM supplemented with 1 mM Phi as the P source showed that two randomly selected hygromycin resistant clones (one per construct), *ccg6*_OPT_-3 and *pki1*_OPT_-6, were also able to use Phi as a P source (Additional file 1: Figure S5a). To determine whether expression of *ptxD* has a detrimental effect on the growth of *T. atroviride*, we measured the radial growth, in a time-course experiment, during 72 h in standard media for two independent transgenic clones for each construct (*ccg6*_OPT_-3, and -5, and *pki1*_OPT_-6 and -5). We found that the four transgenic strains displayed a growth rate comparable to that of the TaWT control with no statistical difference, indicating that their vegetative growth was not affected by the expression of *ptxD* (Additional file 1: Figure S5b). We then selected six *T. atroviride* transgenic lines, three harboring the *ccg6*_OPT_ construct (*ccg6*_OPT_-3, -5 and -6) and three containing the *pki1*_OPT_ construct (*pki*_OPT_-1, -4 and -6), to evaluate their ability to grow in liquid media supplemented with 1 mM Phi as P source. Media supplemented with Pi and lacking a P source were used as controls. After 8 days of incubation, we observed that the six transgenic lines grew as well as the WT control in media containing Pi as a P source, whereas none of the strains grew in media devoid of a P source (Fig. 5a). In media containing Phi as a sole P source the TaWT strain was unable to sustain growth, while the four transgenic strains showed a similar growth as the WT grown in media containing Pi as a P source (Fig. 5a). These transgenic clones were also able to grow in higher Phi concentrations (2, 4 and 5 mM; Additional file 1: Figure S6). To investigate whether the *ccg6* promoter had a similar capacity to drive the expression of *ptxD* as that of P*pki1*, three transgenic clones containing each construct were inoculated in triplicate into flaks containing liquid minimal media supplemented with Phi as a sole P source and the dry weight of the accumulated biomass determined. We found that all the strains expressing *ptxD* accumulate a substantial amount of biomass (between 3.7 and 5.1 mg mL^−1^ DW) in media containing Phi as a sole source of P, whereas the TaWT in the same media did not accumulate a significant amount of biomass (0.23 mg mL^−1^ DW) (Fig. 5b). No statistical difference in growth was detected between the growth of the clones containing the constructs *ccg6*_OPT_ and *pki1*_OPT_. These results indicate that the *ccg6* sequence is functional and acts as a constitutive promoter, as it has the ability to direct a similar level of *ptxD* expression as that directed by the *pki1* promoter, which is reflected in the capacity of the transformants for normally growing in media containing Phi as a sole P source.

To further characterize the transgenic clones, the presence of *ptxD* and presence and level of *ptxD* transcripts, and enzymatic activity were determined in different *ccg6*_OPT_ and *pki1*_OPT_ transgenic clones. Using genomic DNA, we determined by PCR that the *ptxD* gene is present in all the transformants analyzed (Additional file 1: Figure S7). To corroborate that the similar growth observed for *ccg6*_OPT_ and *pki1*_OPT_ strains indeed represents that the *ccg6* promoter can drive the expression of *ptxD* at a similar level, *ptxD* transcript levels were determined by qRT-PCR for both types of transgenic strains. We found that the two promoters lead to the accumulation of similar levels of *ptxD* transcript, although the *ccg6* promoter appears to drive a slightly higher level of expression (Fig. 5c). PTXD enzymatic activity determined through a fluorescence-based method for NADH detection corroborated that both *ccg6*_OPT_ and *pki1*_OPT_ strains had similar levels of activity (Fig. 5d).

### Expression of the *ptxD* gene does not alter the biocontrol properties of *T. atroviride*

To test whether the expression of the *ptxD* gene under either *pki1* or *ccg6* promoter could cause any potential change in the biological characteristics of *T. atroviride*, we conducted a characterization of the transgenic strains evaluating their mycoparasitism and antagonism activities. In order to evaluate the antagonistic activity of transgenic lines against phytopathogenic fungi, we performed confrontation and antibiosis assays between three selected transformants, *ccg6*_OPT_-3, -5, and -6, and *pki1*_OPT_-2, -5, and -6, against *Rhizoctonia solani* AG5 (*Rs*AG5). For the confrontation experiments, we inoculated *R. solani* on one side of the Petri dish and on the other the TaWT or transgenic strains. In these experiments, we observed that when *Rs*AG5 was inoculated alone, it covered at least 80% of the Petri dish at the end of the experiment (Additional file 1: Figure S8, Additional file 2: Table S1). When *R. solani* was inoculated in the same dish with TaWT, its growth was arrested in less than 25% of the Petri dish total area and was overgrown by the *Trichoderma* strain (Additional file 1: Figure S8). TaWT grew effectively and covered more than 75% of the Petri dish total area (Additional file 2: Table S2). Similar results were obtained when the four transgenic strains were confronted with *Rs*AG5; the growth of the phytopathogenic fungi was arrested and was overgrown by the transgenic *ptxD*-*Trichoderma* strains which covered almost two-third of the Petri dish total area with no statistical difference between the TaWT and the transgenics (Additional file 1: Figure S8, Additional file 2: Table S1). Thus, the three transgenic *T. atroviride* strains displayed equivalent antagonism toward the pathogenic fungus to that observed for the TaWT strain.

For the antibiosis assays, TaWT was inoculated on a cellophane membrane that was placed onto the agar media. After 48 h of TaWT growth on the Petri dish, the cellophane membrane was removed to eliminate fungal mycelia and the clean dish was inoculated with *Rs*AG5 to evaluate the effect of the metabolites released by *Trichoderma* into the media. In control dishes in which a clean cellophane membrane was placed, *Rs*AG5 growth was clearly visible covering over 50% of the Petri dish surface at the end of the experiment (Additional file 1: Figure S9, Additional file 2: Table S2). In Petri dishes in which the cellophane membrane had TaWT, the growth of *Rs*AG5 was completely inhibited. When the three *T. atroviride* transgenic strains were tested in this antibiosis assay, we found that all the strains also completely inhibited the growth of *Rs*AG5 (Additional file 1: Figure S9, Additional file 2: Table S2). These results suggest that neither the use of the promoter *ccg6* or the expression of the *ptxD* gene interfere with the biological properties of *T. atroviride*.

### Cultivation of *Trichoderma* in Phi-containing media prevents the growth of contaminant bacteria

**Fig. 6:**
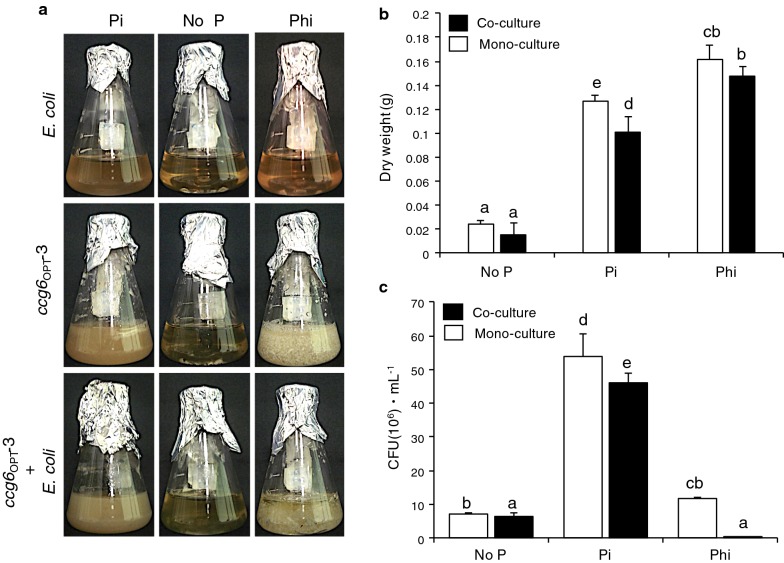
Competition experiments between *T. atroviride* transgenic strain *ccg6*_OPT_-3 and *E. coli*. **a***Trichoderma* transgenic line *ccg6*_OPT_-3 was grown in competition with *E. coli* in modified Vogel’s minimal media supplemented with 4 mM phosphite (Phi) as the only phosphorus (P) source. *E. coli* and *ccg6*_OPT_-3 monocultures were included as controls. Media without P (No P) and with phosphate (Pi) as the P source were used as controls. After 7 days of incubation glass flasks were photographed and **b** mycelia was harvested from the media by filtration with a sterile filter paper, and dry weight (g) determined. **c** CFU mL^−1^ (× 10^6^) was determined in the filtrate as an indication of *E. coli* growth. Data are expressed as mean ± SE, n = 3 (*p *< 0.05). Different letters indicate statistical difference

Contaminant organisms are a major barrier for the establishment of effective bioprocess, as they compete for nutrients and general resources with the organisms of interest and, thus, compromise yield of biomass and yield and quality of the bioproduct. Biological contamination is a serious constrain for the industrial use of yeast strains (i.e. *S. cerevisiae*, *Yarrowia lipolytica*) for biofuels and other fermentative processes, and the *ptxD*/Phi system was proven as an effective strategy for the restriction of contaminations [61–63]. To explore whether *Trichoderma* transformants have the capacity to outcompete contaminating organisms when grown in a Phi-supplemented medium, we designed experiments to simulate *Trichoderma* growth in competition with a bacterial contaminant. With this aim, one of the transgenic *T. atroviride* strains (*ccg6*_OPT_-3) and *E. coli,* as the competitor contaminant, were selected for experimentation. We established co-culture experiments by growing *Trichoderma ccg6*_OPT_-3 and *E. coli* together in VMM supplemented with 4 mM Phi as the sole P source, and No P and Pi as control treatments (Fig. 6a). *E. coli* and *T. atroviride ccg6*_OPT_-3 monocultures grown under the same conditions were also established. For *E. coli*, growth was determined as colony-forming units (CFU) in LB media, whereas *Trichoderma* biomass was measured as mycelia dry weight. Mycelium and *E. coli* cells were separated by filtering in Whatman filter paper.

Under control conditions without a P source, both *E. coli* (less than 10 × 10^6^ CFU mL^−1^) and *ccg6*_OPT_-3 (less 0.025 g DW) showed limited growth above the original inoculum in both monoculture and co-culture conditions (Fig. 6a–c). Under Pi conditions, *ccg6*_OPT_-3 showed biomass production of 0.1263 g DW growing as monoculture and 0.1 g DW when grown in co-culture with *E. coli* (Fig. 6b). *E. coli* achieved a growth of 53 × 10^6^ CFU mL^−1^ growing as monoculture and 46 × 10^6^ CFU growing as co-culture (Fig. 6c). These results indicate that *E. coli* and the transgenic *T. atroviride* have reduced growth when grown in mixed cultures probably resulting from competition for essential nutrients or the release of metabolites that inhibit the growth of each other. When Phi was supplemented as the sole P source, the growth of *E. coli* in monoculture was 5 to 6 times lower than that observed in media containing Pi as a P source and similar to that obtained in media lacking a P source. In the *Ta*/*Ec* mixed culture in media containing Phi as sole P source, *E. coli* CFUs were much lower than those observed when the bacterium was grown alone in media lacking a P source or media containing Phi as P source, indicating that the presence of Phi and an actively growing *T. atroviride* strain have a synergistic negative effect on the growth of *E. coli*. Interestingly, the biomass accumulation of *ccg6*_OPT_-3 under Phi conditions was higher, both as monoculture and in competition with *E. coli*, than that obtained in media containing Pi as a P source. These data show that the engineered strains of *Trichoderma* expressing the *ptxD* gene are able to outcompete the contaminant organism when grown in media containing Phi, and that the system is effective controlling bacterial contaminations.

## Discussion

*Trichoderma* species have gained reputation as biotechnology workhorses for the expression of heterologous and homologous genes for industrial applications and also as a model to study functional genomics in fungi. Constitutive promoters are often preferable to produce recombinant proteins because they are simple to use and do not require external stimulus or agents to activate expression and lead to purer enzyme preparations, reducing production costs at industrial scale.

In this work we report the identification and use of P*ccg6*, a novel constitutive promoter from TaWT, that has similar transcriptional strength as that of the P*pki*1, and the use of PTXD, a phosphite oxidoreductase, as a growth reporter gene that allows real-time comparison of different promoters. *Taccg6* is the orthologue of the *ccg6* gene of *N. crassa* and presents nearly constitutive expression behavior according to our analyses (Fig. 1). Eight *ccgs* genes have been reported and widely studied in this fungal species (*ccg1*, *ccg2*, *ccg4*, *ccg6*, *ccg7*, *ccg8*, *ccg9*, *ccg12*). *Ccg1*, *2*, *4*, *6*, *7* and *9* have been reported to display expression patterns similar to conidiation-specific genes and are regulated by light [64]. However, others including *ccg12*, *7* and *8*, showed no regulation in response to light conditions or other factors inducing developmental changes, suggesting that they could be involved in cellular processes other than conidiation. Indeed, *ccg12* encodes for a copper metallothionein involved in copper storage and detoxification; *ccg7* encodes a glyceraldehyde-3-phosphate dehydrogenase, an important enzyme in gluconeogenesis; and *ccg9* encodes a trehalose synthase probably involved in protecting proteins and membranes under stress conditions [64–66]. In contrast to *ccg6 in N. crassa*, we have not observed circadian behavior in the expression of the *ccg6* gene in *T. atroviride* (*unpublished data*) [27, 48, 49]. According to our analyses, the *Taccg6*-encoded protein belongs to the SED1/SPI1 protein family and is highly similar and conserved between the different fungal species we analyzed. Proteins belonging to the SPE1/SPI1 family have been reported to be important cell wall components in *S. cerevisiae* and to play a role during stress conditions [67]. Further analyses are needed to gain insight about the biological function of CCG6 in *Trichoderma* and other fungal species.

A comparative analysis of 600 bp promoter fragment of *T. atroviride*, *T. reesei*, and *T. asperellum ccg6* orthologues suggest the presence of common DNA motifs that resembled the binding sequences present in the promoter of a *Sed1* gene in *S. cerevisiae* [52], suggesting a similar regulation. Other TFs present in *ccg6* promoter are Nrg1, Mig1, Mig2, Mig3, and Bas1, Met31, Met32 involved in the metabolism of carbohydrate and aminoacids in *S. cerevisiae* [68]. Interestingly, an important group of motifs including Haa1p, Ume6, Yrr1, and Stb5, are involved in regulating stress responses which correlate with the stress conditions used to generate the RNA-seq databases we analyzed [68]. A smaller group of motifs are also related to the general cell growth (Rap1, Ash1, Cbf1) as reported for *S. cerevisiae* [69]. None of the putative TF binding motifs identified in P*cc6* resembled the activating clock element, which is required for the rhythmic transcription of *ccg*-*2* in *N. crassa* [70]. However, whether the DNA motifs identified in this work effectively correspond to motifs regulating the transcriptional activity of P*ccg6* remains to be further explored.

Using a 600 bp fragment upstream of the predicted transcriptional start site of the *Taccg6* promoter, we successfully expressed the *ptxD* gene in *T. atroviride* achieving similar expression levels as those achieved using the *pki1* promoter. The fungal cells expressing the phosphite oxidoreductase were able to use Phi as a sole source of P. This capacity resulted in a dominant, visible, and easily scorable phenotype, in both liquid and solid media, that provides a growth marker tool to assess the functionality of predicted promoter sequences by growing the engineered *Trichoderma* in a simple media supplemented with Phi. Moreover, the phenotype is robust and stable as the transformants were able to grow normally under high concentrations of this novel source of P. The enzymatic reaction catalyzed by PTXD is simple and involves the direct conversion of Phi into Pi, using NAD^+^ as cofactor. The products of the reaction, Pi and NADH, are both ubiquitous in living cells, therefore, we do not anticipate any interference in the general metabolism of the host organisms due to the incorporation of this new metabolic pathway. *Trichoderma ptxD*-transformants harboring *ccg6*_OPT_ or *pki1*_OPT_ gene constructs did not show negative effects of growth or phenotype, suggesting that expression of PTXD in *Trichoderma* has no negative effects on its physiology and that the transgenic strains have similar growth kinetics on Pi- and Phi-containing media. We observed an effective growth inhibition of Phi in three different *Trichoderma* species (TaWT, Tv, Tr), either when conidia or mycelium plugs were used as inoculum. Therefore, the PTXD/Phi system is probably also applicable to other *Trichoderma* species and perhaps other unrelated fungal species. The observed inhibitory effect on the growth of *Trichoderma* species was dependent on the Phi concentration in the media and influenced by Pi reserves probably present in the inoculum, supporting previous finding showing that Phi enters fungal cells and compete with Pi using the same transport systems [71, 72].

*Trichoderma* species are widely used at industrial level in the manufacture of a variety of pharmaceuticals, chemicals, and enzyme products [73]. Most of the enzymes required by the food industries, for example, are produced by recombinant strategies in *T. reesei*, typically cultured by submerged fermentation, and receive the generally recognized as safe (GRAS) status by the U.S. Food and Drug Administration (FDA) [73, 74]. Therefore, they have to comply with safety studies to assess not only the enzyme and the strain safety but also the absence of viable contaminant cells, toxins and secondary metabolites. Manufacture of safe and high-quality enzyme preparations requires the culture of pure strains throughout the process, requiring the implementation of expensive and complex contamination control measures (i.e. antibiotic dosage), the use of sanitary stainless-steel tanks and piping and steam sterilization of the growth media and accessories, which increase operation costs [74]. The implementation of these measures in *T. reesei* is extremely important for the production of enzyme preparations intended for food processing, as clearly manifested by companies that state that any batch must be rejected if contamination is detected (i.e. DuPont, Novozyme, AB Enzyme) [9]. In some cases, antibiotics are used to prevent bacterial contamination [73–75], however, the use of antibiotics has been under increased scrutiny by the consumers and is not a well-accepted in the food industry. In this work, we report that the *ptxD*-expressing strain outcompetes a bacterial contaminant strain when cultured in liquid Phi-based media (Fig. 6), suggesting that the Phi metabolism could be a promising strategy to control contamination during enzyme production in *Trichoderma* and, thus reducing the need for using other practices to prevent contaminations such as the use of antibiotics and tank and media sterilization. We previously reported that expression of PTXD plants and microalgae allowed the creation of a highly selective environment that favors the development of the engineered organisms while compromising the growth of complex mixtures of weedy organisms (plants, microalgae, fungi and bacteria) unable to metabolize Phi [58, 59, 76, 77]. Moreover, the use of the PTXD/Phi was shown to be an efficient strategy to control contaminant organisms (i.e. *Kluyveromyces marxianus* CBS 6556, *S. cerevisiae* Ethanol Red) during batch fermentation with Phi-metabolizing *S. cerevisiae* and *Y. lipolytica* strains using low-cost feedstocks [61].Thus, the *ptxD*/Phi system holds promise to decrease cost of enzymes manufacture using *Trichoderma*, as antibiotics or reactor sterilization are expensive in comparison to Phi salts; a search in the Alibaba web site indicates that 1 kg of monopotassium phosphite (98% purity) costs around US$1.5. Moreover, Phi is stable and when properly stored can last for years compared to the relatively short shelf life of antibiotics. Since Phi has been approved by the FDA for use as a fungicide for application in agriculture and as a food additive, the *ptxD*/Phi system represents a safer and less expensive alternative to control contamination for the industrial production of *Trichoderma* and potentially other filamentous fungi. However, an economic assessment under realistic cultivation conditions using complex carbon sources is necessary.

Although not formally addressed in this report, preliminary experiments in our laboratory suggest that 1 mM Phi can be used as an effective selective agent for the transformation of *T. atroviride* protoplast. Therefore, it seems feasible to eliminate the use of antibiotic resistance genes, which have little acceptance by consumers, to genetically engineer different species of *Trichoderma*.

## Conclusions

We identified and tested a new constitutive promoter, *ccg6*, for expression of homologous and heterologous proteins in *T. atroviride*. A 600 bp sequence upstream the transcription start site of the *T. atroviride ccg6* gene was efficient in driving the expression of *ptxD*, a phosphite oxidoreductase-encoding gene of bacterial origin, to similar levels as those achieved using the *pki1* promoter. The expression of PTXD does not compromise *Trichoderma* growth and biological properties, and therefore, resulted in an effective and visible phenotype to evaluate transcriptional activity of regulatory sequences. In addition, the Phi-metabolism provided *T. atroviride* a competitive advantage to outgrow bacterial contaminants when fed with Phi. The use of P*ccg6* for homologous and heterologous expression of proteins of industrial interest and the use of PTXD as a growth marker holds great potential for assessing activity of other promoters and for biotechnological applications as a contamination control system.

## Supplementary information

**Additional file 1.** Additional figure and legends supporting the results described in the text.

**Additional file 2.** Additional tables and legends supporting the results described in the text.

## Data Availability

RNA-seq data sets analyzed to identify the *ccg6* gene are available at the European Nucleotide Archive (https://www.ebi.ac.uk/ena/) and have been partially published in [27, 45, 48, 49]. Sequences of the *ccg6* promoter and the *ptxD* gene codon-optimized for *T. atroviride* were submitted to the GenBank database under accession numbers MK887357 and MN434083, respectively.

## Abbreviations

*ccg*: Clock-controlled gene
CFU: Colony forming units
DW: Dry weight
FDA: U.S. Food and Drug Administration
GRAS: Generally recognized as safe
P: Phosphorus
P*ccg6*: Promoter of the *clock*-*controlled gene*-*6* from *Trichoderma atroviride* IMI 206040
PDA: Potato dextrose agar
PDB: Potato dextrose broth
Phi: Monopotassium phosphite
Pi: Monopotassium phosphate (orthophosphate)
PTXD: Phosphite oxidoreductase
*ptxD*: Phosphite oxidoreductase-encoding gene
*Rs*AG5: *Rhizoctonia solani* AG5
SED1/SPI1: Suppression of Exponential Defect1/Stationary Phase Induced1
*Taccg6*: *Clock*-*controlled gene*-*6* from *Trichoderma atroviride* IMI 206040
TaWT: *Trichoderma atroviride* IMI 206040
TF: Transcription factor
Tr: *Trichoderma reesei* QM6a
Tv: *Trichoderma virens* gV29-8
VMM: Volgel’s minimal medium

## References

[1] Schuster A, Schmoll M (2010). Biology and biotechnology of *Trichoderma*. Appl Microbiol Biotechnol.

[2] Félix CR, Ferreira Noronha E, Miller RNG, Gupta VK, Schmoll M, Herrera-Estrella A, Upadhyay RS, Druzhinina I, Tuohy MG (2014). *Trichoderma:* a dual function fungi and their use in the wine and beer industries. Biotechnology and biology of *Trichoderma*.

[3] Kunamneni A, Plou FJ, Alcalde M, Ballesteros A, Gupta VK, Schmoll M, Herrera-Estrella A, Upadhyay RS, Druzhinina I, Tuohy MG (2014). *Trichoderma* enzymes for food industries. Biotechnology and biology of Trichoderma.

[4] Puranen T, Alapuranen M, Vehmaanperä J, Gupta VK, Schmoll M, Herrera-Estrella A, Upadhyay RS, Druzhinina I, Tuohy MG (2014). *Trichoderma* enzymes for textile industries. Biotechnology and biology of *Trichoderma*.

[5] Juhász T, Kozma K, Szengyel Z, Réczey K (2003). Production of β-glucosidase in mixed culture of *Aspergillus niger* BKMF 1305 and *Trichoderma reesei* RUT-C30. Food Tech Biotech..

[6] Ahamed A, Vermette P (2008). Enhanced enzyme production from mixed cultures of *Trichoderma reesei* RUT-C30 and *Aspergillus niger* LMA grown as fed batch in a stirred tank bioreactor. Biochem Eng J.

[7] Gottschalk LM, Alves-Oliveira R, Pinto da Silva-Bon E (2010). Cellulases, xylanases, β-glucosidases and ferulic acid esterease produced by *Trichoderma* and *Aspergillus* act synergistically in the hydrolysis of sugarcane bagasse. Biochem Eng J.

[8] Ma L, Zhang J, Zou G, Wang C, Zhou A (2011). Improvement of cellulose activity in *Trichoderma reesei* by heterologous expression of a beta-glucosidase gene from *Penicillium decumbens*. Enzyme Microb Technol.

[9] U.S Food and Drug Administration. https://search.usa.gov/search?query=Trichoderma+produced+enzymes&affiliate=fda1. Accessed 23 Oct 2019.

[10] Zou G, Shi S, Jiang Y, van den Brink J, de Vries RP, Chen L (2012). Construction of a cellulase hyper-expression system in *Trichoderma reesei* by promoter and enzyme engineering. Microb Cell Fact.

[11] Fitz E, Wanka F, Seiboth B (2018). The promoter toolbox for recombinant gene expression in *Trichoderma reesei*. Front Bioeng Biotechnol.

[12] Rantasalo A, Landowski CP, Kuivanen J, Korppoo A, Reuter L, Koivistoinen O (2018). A universal gene expression system for fungi. Nucleic Acids Res.

[13] Singh A, Taylor IILE, Vander Wall TA, Linger J, Himmel ME, Podkaimer K (2015). Heterologous gene expression in *Hypocrea jecorina*: a historical perspective and new developments. Biotechnol Adv.

[14] Filipek J, Mach RL, Herzog P, Sowka S, Kubicek CP, Kurzatkowski W, Törrönen A (1996). Glucose-induced secretion of *Trichoderma reesei* xylanases. Appl Environ Microbiol.

[15] Nakari-Setala T, Penttila M (1995). Production of *Trichoderma reesei* cellulases on glucose-containing media. Appl Environ Microbiol.

[16] Delgado-Jarana J, Pintor-Toro J, Benitez T (2000). Overproduction of b-1,6-glucanase in *Trichoderma harzianum* is controlled by extracellular acidic proteases and pH. Biochim Biophys Acta.

[17] He R, Zhang C, Guo W, Wang L, Zhang D, Chen S (2013). Construction of a plasmid for heterologous protein expression with a constitutive promoter in *Trichoderma reesei*. Plasmid.

[18] Li J, Wang J, Wang S, Xing M, Yu S, Liu G (2012). Achieving efficient protein expression in *Trichoderma reesei* by using strong constitutive promoters. Microb Cell Fact.

[19] Balcázar-López E, Méndez-Lorenzo LH, Batista-García RA, Esquivel-Naranjo U, Ayala M, Kumar VV, Savary O (2016). Xenobiotic compounds degradation by heterologous expression of *Trametes sanguineus* laccase in *Trichoderma atroviride*. PLoS ONE.

[20] Kurzatkowski W, Torronen A, Filipek J, Mach RL, Herzog P, Sowka S, Kubicek CP (1996). Glucose-induced secretion of *Trichoderma reesei* xylanases. Appl Environ Microbiol.

[21] Mach RL, Schindler M, Kubicek CP (1994). Transformation of *Trichoderma reesei* based on hygromycin B resistance using homologous expression signals. Curr Genet.

[22] Zohar-Perez C, Chet I, Nussinovitch A (2004). Unexpected distribution of immobilized microorganisms within alginate beads. Biotechnol Bioeng.

[23] Wang W, Shi XY, Wei DZ (2014). Light-mediated control of gene expression in filamentous fungus *Trichoderma reesei*. J Microbiol Methods.

[24] Nakari T, Alatalo TE, Penttila ME (1993). Isolation of *Trichoderma reesei* genes highly expressed on glucose-containing media: characterization of the *tef1* gene encoding translation elongation factor 1. Gene.

[25] Metz B, Seidl-Seiboth V, Haarmann T, Kopchinskiy A, Lorenz P, Seiboth B (2011). Expression of biomass-degrading enzymes is a major event during conidium development in *Trichoderma reesei*. Eukaryot Cell.

[26] Herrera-Estrella A, Goldman GH, Van Montagu M (1990). High-efficiency transformation system for the biocontrol agents, *Trichoderma* spp. Mol Microbiol..

[27] Cetz-Chel JE, Balcazar-Lopez E, Esquivel EU, Herrera-Estrella A (2016). The *Trichoderma atroviride* putative transcription factor Blu7 controls light responsiveness and tolerance. BMC Genomics..

[28] Medina-Castellanos E, Villalobos-Escobedo JM, Riquelme M, Read ND, Abreu-Goodger C, Herrera-Estrella A (2018). Danger signals activate a putative innate immune system during regeneration in a filamentous fungus. PLoS Genet.

[29] Bray NL, Pimentel H, Melsted P, Pachter L (2016). Near-optimal probabilistic RNA-seq quantification. Nat Biotechnol.

[30] Soneson C, Love MI, Robinson MD (2016). Differential analyses for RNA-seq: transcript-level estimates improve gene-level inferences. F1000Research..

[31] Smid M, Coebergh van den Braak RRJ, van de Werken HJG (2018). Gene length corrected trimmed mean of M-values (GeTMM) processing of RNA-seq data performs similarly in intersample analyses while improving intrasample comparisons. BMC Bioinf.

[32] Katoh K, Misawa K, Kuma K, Miyata T (2002). MAFFT: a novel method for rapid multiple sequence alignment based on fast Fourier transform. Nucleic Acids Res.

[33] ExPASY. Bioinformatic Resource Portal. BOXSHADE server version 3.21. http://www.expasy.org.

[34] Robinson O, Dylus D, Dessimoz C (2016). Phylo.io: interactive viewing and comparison of large phylogenetic trees on the web. Mol Biol Evol.

[35] InterProScan Server. Classification of protein families. http://www.ebi.ac.uk/InterProSacan.

[36] Almagro Armenteros JJ, Tsirigos KD, Sonderby CK, Petersen TN, Winther O, Brunak S (2019). SignalP 5.0 5.0: improved signal peptide predictions across the tree of life using deep neural networks. Nat Biotechnol.

[37] Bailey TL, Bodén M, Buske FA, Frith M, Grant CE, Clementi L, Ren J, Li WW, Noble WS (2009). MEME SUITE: tools for motif discovery and searching. Nucleic Acids Res.

[38] Gupta S, Stamatoyannopolous JA, Bailey T, Stafford-Noble W (2007). Quantifying similarity between motifs. Genome Biol.

[39] Vogel HJ (1956). Microbial genetics. Bulletin..

[40] Khokhar ZU, Syed QU, Wu J, Athar MA (2014). On-site cellulase production by *Trichoderma reesei* 3EMS35 mutant and same vessel saccharification and fermentation of acid treated wheat straw for ethanol production. Excli J..

[41] Mutawila C, Vinale F, Halleen F, Lorito M, Mostert L (2016). Isolation, production and in vitro effects of the major secondary metabolite produced by *Trichoderma* species used for the control of grapevine trunk diseases. Plant Pathol.

[42] Seidl V, Song L, Lindquist E, Gruber S, Koptchinskiy A, Zeilinger S (2009). Transcriptomic response of the mycoparasitic fungus *Trichoderma atroviride* to the presence of a fungal prey. BMC Genomics..

[43] Vargas WA, Mandawe JC, Kenerley CM (2009). Plant-derived sucrose is a key element in the symbiotic association between trichoderma virens and maize plants. Plant Physiol.

[44] Rueden CT, Schindelin J, Hiner MC, DeZonia BE, Walter AE, Arena ET, Eliceiri KW (2017). Image J2: imageJ for the next generation of scientific image data. BMC Bioinf.

[45] Hernandez-Oñate MA, Esquivel-Naranjo EU, Mendoza-Mendoza A, Stewart A, Herrera-Estrella AH (2012). An injury-response mechanism conserved across kingdoms determines entry of the fungus *Trichoderma atroviride* into development. Proc Natl Acad Sci USA.

[46] Casas-Flores S, Rios-Momberg M, Rosales-Saavedra T, Martínez-Hernández P, Olmedo-Monfil V, Herrera-Estrella A (2006). Cross talk between a fungal blue-light perception system and the cyclic AMP signaling pathway. Eukaryot Cell.

[47] Mukherjee PK, Horwitz BA, Herrera-Estrella A, Schmoll M, Kenerley CM (2013). *Trichoderma* research in the new era. Annu Rev Phytopathol.

[48] Carreras-Villaseñor N, Esquivel-Naranjo EU, Villalobos-Escobedo JM, Abreu-Goodger C, Herrera-Estrella A (2013). The RNAi machinery regulates growth and development in the filamentous fungus *Trichoderma atroviride*. Mol Microbiol.

[49] Schmoll M, Dattenböck C, Carreras-Villaseñor N, Mendoza-Mendoza A, Tisch D, Alemán MI (2016). The genomes of three uneven siblings: footprints of the lifestyles of three *Trichoderma* species. Microbiol Mol Biol Rev.

[50] Kersey PJ, Allen JE, Allot A, Barba M, Boddu S, Bolt BJ (2018). Ensembl Genomes 2018: an integrated omics infrastructure for non-vertebrate species. Nucleic Acids Res..

[51] Simoes T, Mira NP, Fernandes AR, Sa-Correia I (2006). The *SPI1* gene, encoding a glycosylphosphatidylinositol-anchored cell wall protein, plays a prominent role in the development of yeast resistance to lipophilic weak- acid food preservatives. Appl Environ Microbiol.

[52] Cardona F, Del Olmo ML, Aranda A (2012). Phylogenetic origin and transcriptional regulation at the post-diauxic phase of SPI1, *Saccharomyces cerevisiae*. Cell Mol Bio Lett..

[53] Causton HC, Ren B, Koh SS, Harbison CT, Kanin E, Jennings EG, Lee TI, True HL, Lander ES, Young RA (2001). Remodeling of yeast genome expression in response to environmental changes. Mol Biol Cell.

[54] Vachova L, Devaux F, Kucerova H, Ricicova M, Jacq C, Palkova Z (2004). Sok2p transcription factor is involved in adaptive program relevant for long term survival of *Saccharomyces cerevisiae* colonies. J Biol Chem.

[55] Chua G, Morris QD, Sopko R, Robinson MD, Ryan O, Chan ET, Frey BJ, Andrews BJ, Boone C, Hughes TR (2006). Identifying transcription factor functions and targets by phenotypic activation. Proc Natl Acad Sci USA.

[56] Teixeira MC, Monteiro PT, Palma M, Costa C, Godinho CP, Pais P, Cavalheiro M, Antunes M, Lemos A, Pedreira T, Sá-Correia I (2018). YEASTRACT, an upgraded database for the analysis of transcription regulatory networks in *Saccharomyces cerevisiae*. Nucl Acids Res..

[57] White AK, Metcalf WW (2007). Microbial metabolism of reduced phosphorus compounds. Annu Rev Microbiol.

[58] López-Arredondo DL, Herrera-Estrella L (2012). Engineering phosphorus metabolism in plants to produce a dual fertilization and weed control system. Nat Biotechnol.

[59] Loera-Quezada MM, Leyva-González MA, Velázquez-Juárez G, Sánchez-Calderón L, Do Nascimento M, Lopez-Arredondo D, Herrera-Estrella L (2016). A novel genetic engineering platform for the effective management of biological contaminants for the production of microalgae. Plant Biotechnol J.

[60] Berkowitz O, Pearse SJ, Lambers H, Finnegan PM, Hardy GE, Brien PA (2011). An enzymatic fluorescent assay for the quantification of phosphite in a microtiter plate format. Anal Biochem.

[61] Shaw AJ, Lam FH, Hamilton M, Consiglio A, MacEwen K, Brevnova EE (2016). Metabolic engineering of microbial competitive advantage for industrial fermentation processes. Science.

[62] Westfall PJ, Gardner TS (2011). Industrial fermentation of renewable diesel fuels. Curr Opin Biotechnol.

[63] Musoni M, Destain J, Thonart P, Bahama J-B, Delvigne F (2015). Bioreactor design and implementation strategies for the cultivation of filamentous fungi and the production of fungal metabolites: from traditional methods to engineered systems. Biotechnol Agron Soc Environ.

[64] Bell-Pedersen D, Shinohara M, Loros J, Dunlap JC (1996). Circadian clock-controlled genes isolated from *Neurospora crassa* are late night to early morning specific. Proc Natl Acad Sci USA.

[65] Shinohara ML, Correa A, Bell-Pedersen D, Dunlap JC, Loros JJ (2002). *Neurospora* Clock-Controlled Gene 9 (*ccg*-*9*) encodes trehalose synthase: circadian regulation of stress responses and development. Eukaryot Cell.

[66] Shinohara ML, Loros JJ, Dunlap JC (1998). Glyceraldehyde-3-phosphate dehydrogenase is regulated on a daily basis by the circadian clock. J Biol Chem.

[67] Hagen L, Ecker M, Lagorce A, Francois JM, Sestak S, Rachel R (2004). Sed1p and Srl1p are required to compensate for cell wall instability in *Saccharomyces cerevisiae* mutants defective in multiple GPI-anchored mannoproteins. Mol Microbiol.

[68] Zhu C, Byers KJ, McCord RP, Shi Z, Berger MF, Newburger DE (2009). High-resolution DNA-binding specificity analysis of yeast transcription factors. Genome Res.

[69] Lieb JD, Liu X, Botstein D, Brown PO (2001). Promoter-specific binding of Rap1 revealed by genome-wide maps of protein-DNA association. Nat Genet.

[70] Bell-Pedersen D, Dunlap JC, Loros JJ (1996). Distinct cis-acting elements mediate clock, light, and developmental regulation of the *Neurospora crassa eas* (*ccg*-*2*) gene. Mol Cell Biol.

[71] Coffey MD, Bower LA (1984). *In vitro* variability among isolates of eight *Phytophthora* species in response to phosphorous acid. Phytopathol..

[72] Griffit JM, Akins LA, Grant BR (1989). Properties of the phosphate and phosphite transport system of *Phytophthora palmivora*. Arch Microbiol.

[73] Paloheimo M, Haarmann T, Mäkinen S, Vehmaanperä J, Schmoll M, Dattenböck C (2016). Production of industrial enzymes in *Trichoderma reesei*. Gene expression systems in fungi: advancements and applications fungal biology.

[74] Sewalt V, Shanahan D, Gregg L, Marta JL, Carrillo R (2016). The generally recognized as safe (GRAS) process for industrial microbial enzymes. Ind Biotechnol.

[75] Raveendran S, Parameswaran B, Ummalyma SB, Abraham A, Mathew AK, Madhavan A (2018). Applications of microbial enzymes in food industry. Food Technol Biotechnol.

[76] Heuer S, Gaxiola R, Schilling R, Herrera-Estrella L, López-Arredondo D, Wissuwa M (2017). Improving phosphorus efficiency: a complex trait with emerging opportunities. Plant J..

[77] Pandeya D, López-Arredondo DL, Janga MR, Campbell LM, Estrella-Hernández P, Bagavathiannan MV (2018). Selective fertilization with phosphite allows unhindered growth of cotton plants expressing the *ptxD* gene while suppressing weeds. Proc Natl Acad Sci USA.

