# Supplementary material for: Assessment of the ptxD gene as a growth and selective marker in Trichoderma atroviride using Pccg6, a novel constitutive promoter
Source: Microb Cell Fact. 2020 Mar 18;19:69. doi: 10.1186/s12934-020-01326-z (PMC7081547; doi:10.1186/s12934-020-01326-z)
Supplement: Supplementary file 1 — Additional file 1. Additional figure and legends supporting the results described in the text. [file 12934_2020_1326_MOESM1_ESM.pdf]

Assessment of the *ptxD* gene as a growth and selective marker in *Trichoderma atroviride* using P*ccg6*, a novel constitutive promoter

Nohemí Carreras-Villaseñor[1,+], José Guillermo Rico-Ruiz[1,§], Ricardo M. Chávez Montes[3], Lenin Yong-Villalobos[3], José Fabricio López-Hernández[2,†], Pedro Martínez-Hernández[2], Luis Herrera-Estrella[2,3], Alfredo Herrera-Estrella[2], Damar López-Arredondo[1,3,*]

[1]StelaGenomics México, S de RL de CV, Av. Camino Real de Guanajuato s/n, 36821, Irapuato, Guanajuato, Mexico.

[2]Laboratorio Nacional de Genómica para la Biodiversidad, Unidad de Genómica Avanzada del Centro de Investigación y de Estudios Avanzados del Instituto Politécnico Nacional, Km 9.6 carretera Irapuato León, 36500, Irapuato, Guanajuato, Mexico.

[3]Institute of Genomics for Crop Abiotic Stress Tolerance, Texas Tech University, 79409, Lubbock, Texas, USA.

*For correspondence. Email: Damar.Lopez-Arredondo@ttu.edu; Tel. 8068343364, orcid: https://orcid.org/0000-0001-7389-3143

[+]Present address: Red de Estudios Moleculares Avanzados, Instituto de Ecología A.C., 91070, Xalapa, Mexico.

[§]Present address: Laboratorio Nacional de Genómica para la Biodiversidad, Unidad de Genómica Avanzada del Centro de Investigación y de Estudios Avanzados del Instituto Politécnico Nacional, Km 9.6 carretera Irapuato León, 36500, Irapuato, Mexico.

[†]Present address: Stowers Institute for Medical Research, Kansas City, MO, 64110, USA.

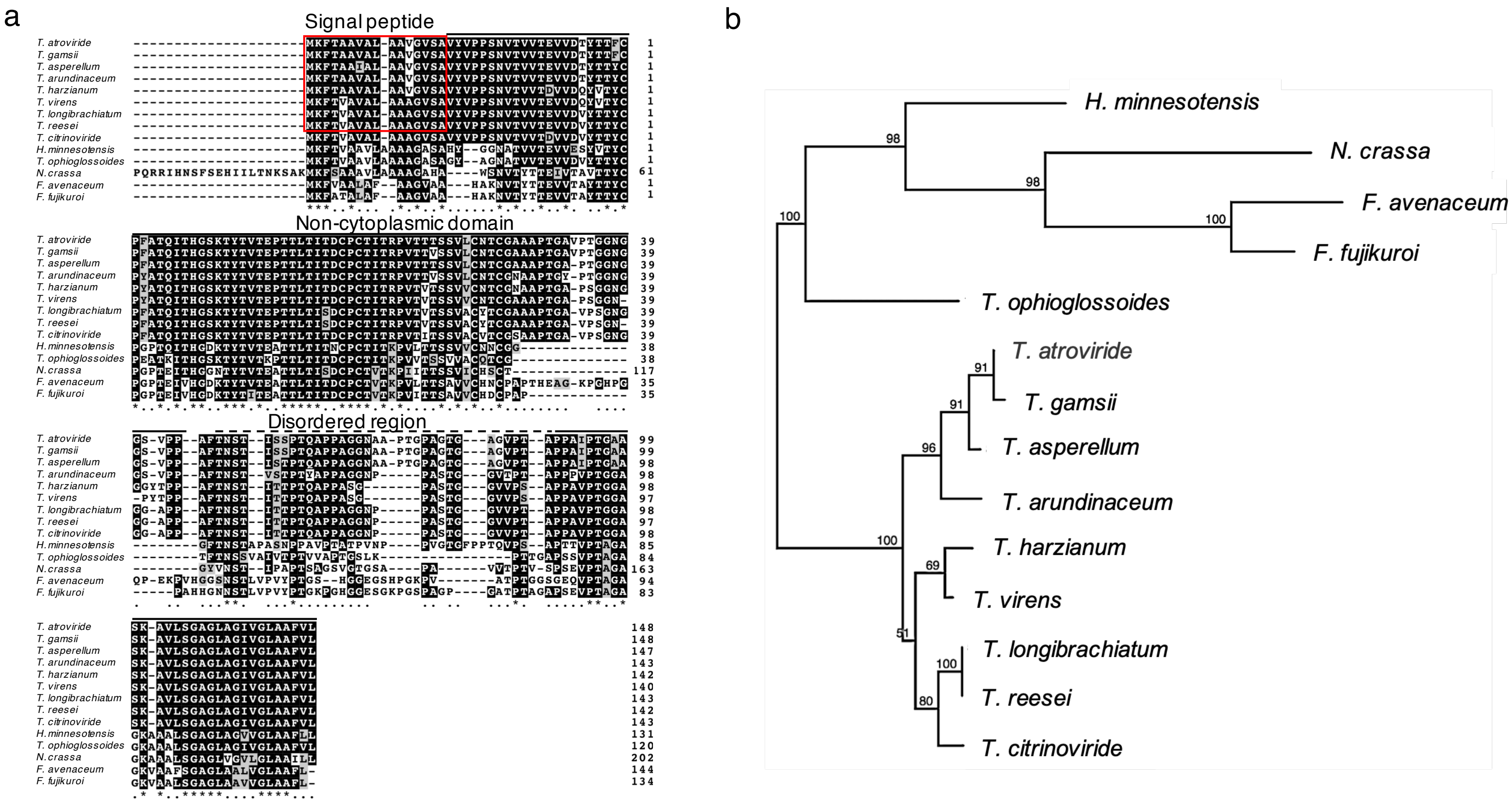

Figure S1. Sequence alignments and phylogenetic tree of CGG6 proteins encoded by *ccg6* orthologues. a) Multiple sequence alignment of the predicted CGG6 protein in *T. atroviride* with ortholog sequences from other fungal species using MAFFT. The conserved residues are shaded in black and equivalent residues are shaded in gray. Consensus symbols means: “*” fully conserved residues; “:” conserved substitutions; “.” semi-conserved substitutions. Analyses of the *Trichoderma* CCG6 proteins using the InterProScan and SignalP servers [35, 36] suggest the presence of a putative signal peptide (red frame) and a non-cytoplasmic domain and a disordered region in the protein. b) Phylogenetic tree of the protein sequences CGG6 homologues from different fungal species constructed using the Neighbor-Joining method. Bootstrap values (%), indicated at the nodes, were obtained from 100 bootstrap replicates. NCBI accession numbers are in the Materials and Methods section.

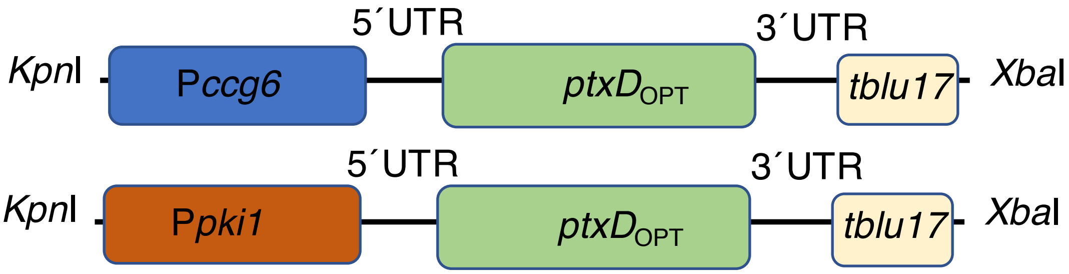

Figure S2. Illustration of DNA constructs generated to express the codon-optimized *ptxD* gene from *Pseudomonas stutzeri* WM88 under control of the *ccg6* and *pki1* promoters. *ptxD* was optimized according the codon usage of *Trichoderma atroviride* and fused to the 5'UTR and 3'UTR of *cbh1* gene of *Trichoderma reesei*. Both constructs were flanked by the *Kpn*I and *Xba*I restriction sites to be cloned in the multiple cloning site of pCB1004 [19].

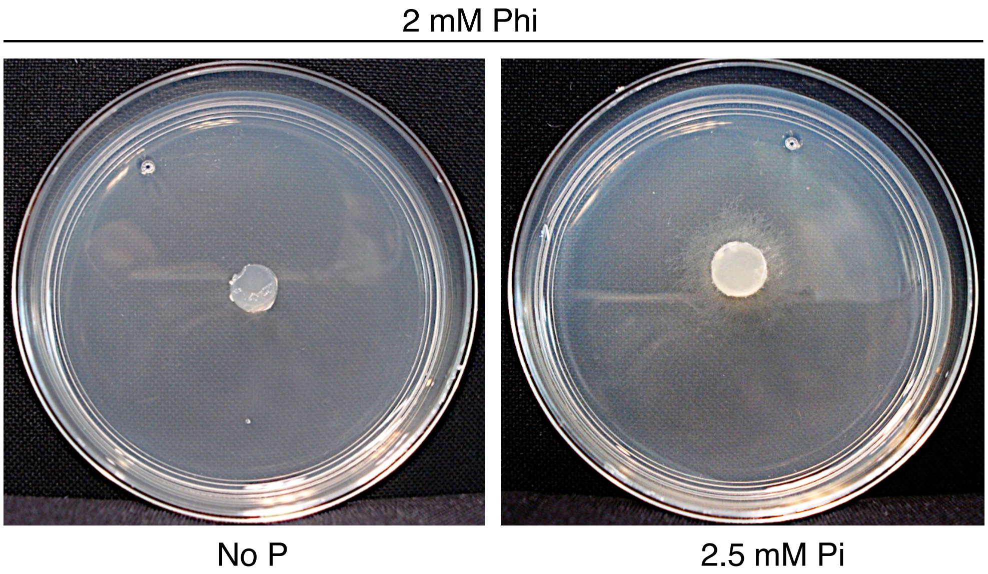

Figure S3. Growth of *T. atroviride* in phosphite media when inoculum is produced in low phosphate-media. *T. atroviride* IMI 206040 fresh mycelium plugs produced using Vogel´s minimal media without a phosphorus source (No P) and with 2.5 mM phosphate (Pi) were inoculated in modified Vogel´s minimal media with 2 mM phosphite (Phi) as the P source. Petri dishes were photographed after 15 days of incubation.

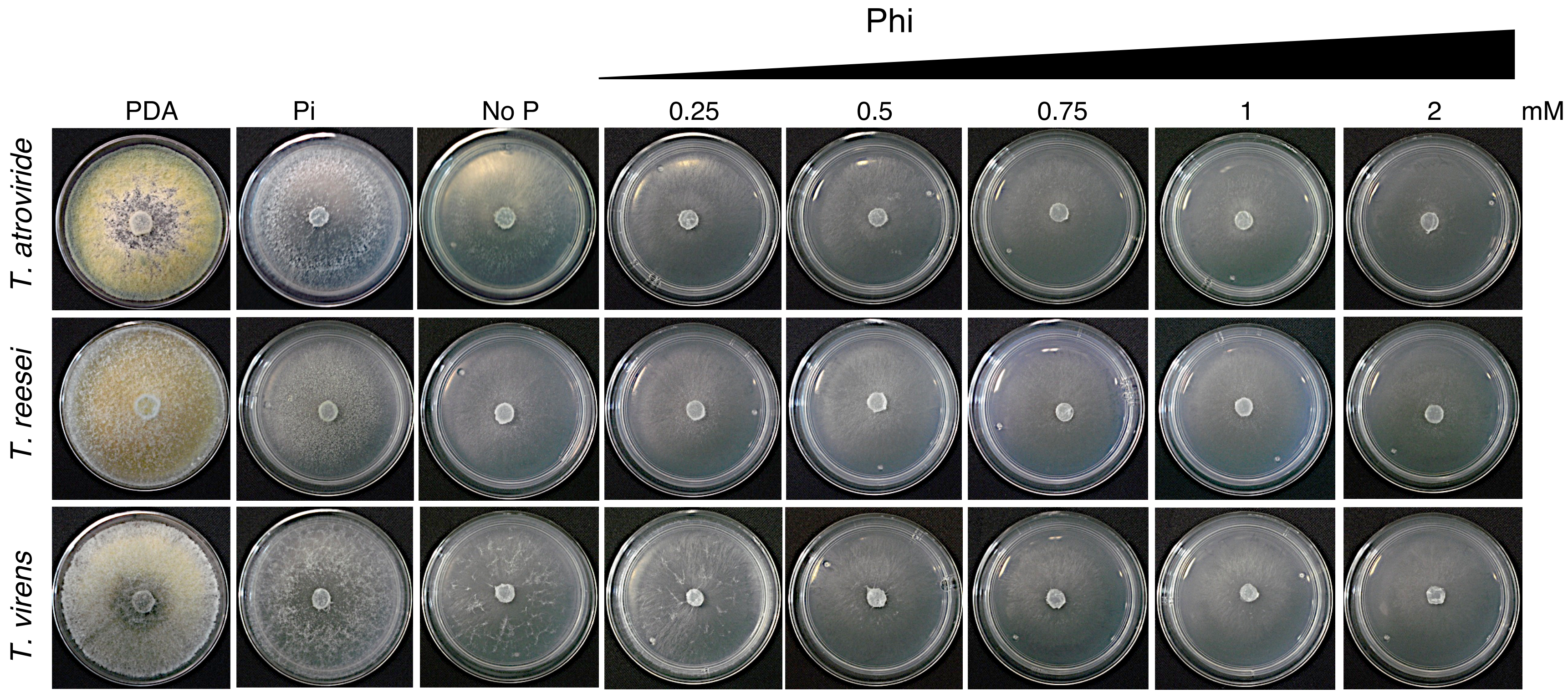

Figure S4. Effect of phosphite in the growth of *T. virens*, *T. reesei and T. atroviride*. *T. atroviride* IMI 206040, *T. reesei* QM6a, and *T. virens* Gv29-8 mycelium plug was inoculated in modified solid Vogel's minimal media supplemented with different concentrations of phosphite (Phi; 0.25, 0.5, 0.75, 1 and 2 mM) as the sole phosphorus (P) source. Media without P (No P), with phosphate (Pi) as the P source, and PDA were used as controls.

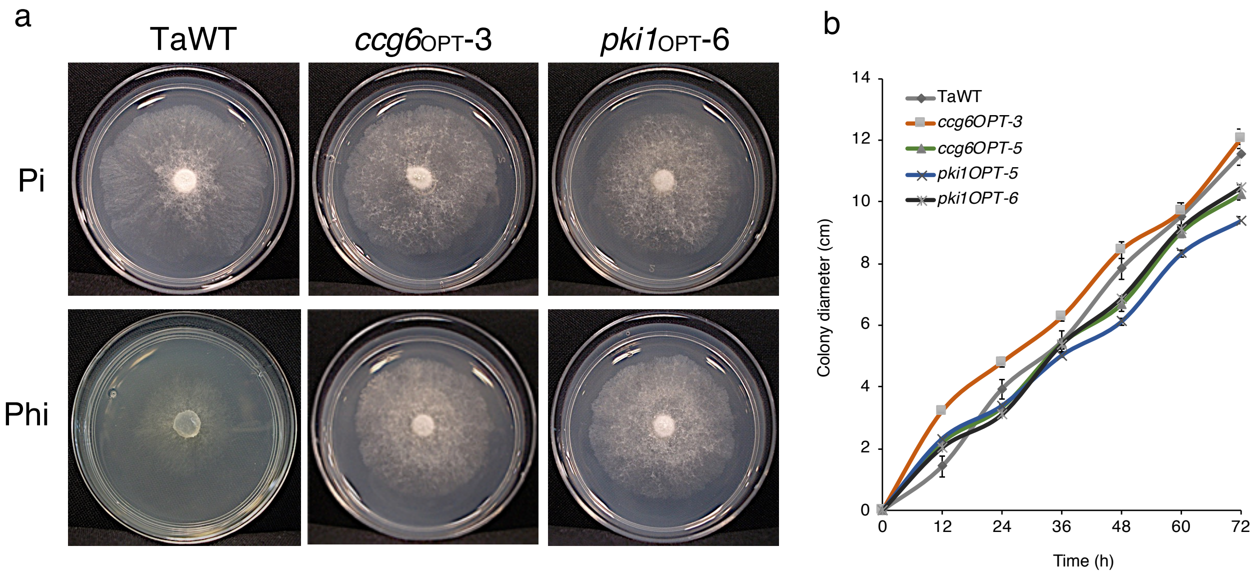

Figure S5. Growth of *T. atroviride* transgenic strains in media with phosphite and growth kinetics on standard conditions. a) Mycelium plugs of *T. atroviride* transformants *ccg6*OPT-3 and *pki1*OPT-6 and *T. atroviride* IMI 206040 (TaWT) were grown in solid modified Vogel's minimal media supplemented with 1 mM phosphite (Phi) as the sole phosphorus source. Media with phosphate (Pi) as the P source was used as control. b) Radial growth (cm) of four *Trichoderma* transformants (*ccg6*OPT-3 and -5, and *pki1*OPT-5 and -6) harboring *ccg6*OPT and *pki1*OPT constructs grown on standard PDA media, every 12 hours.

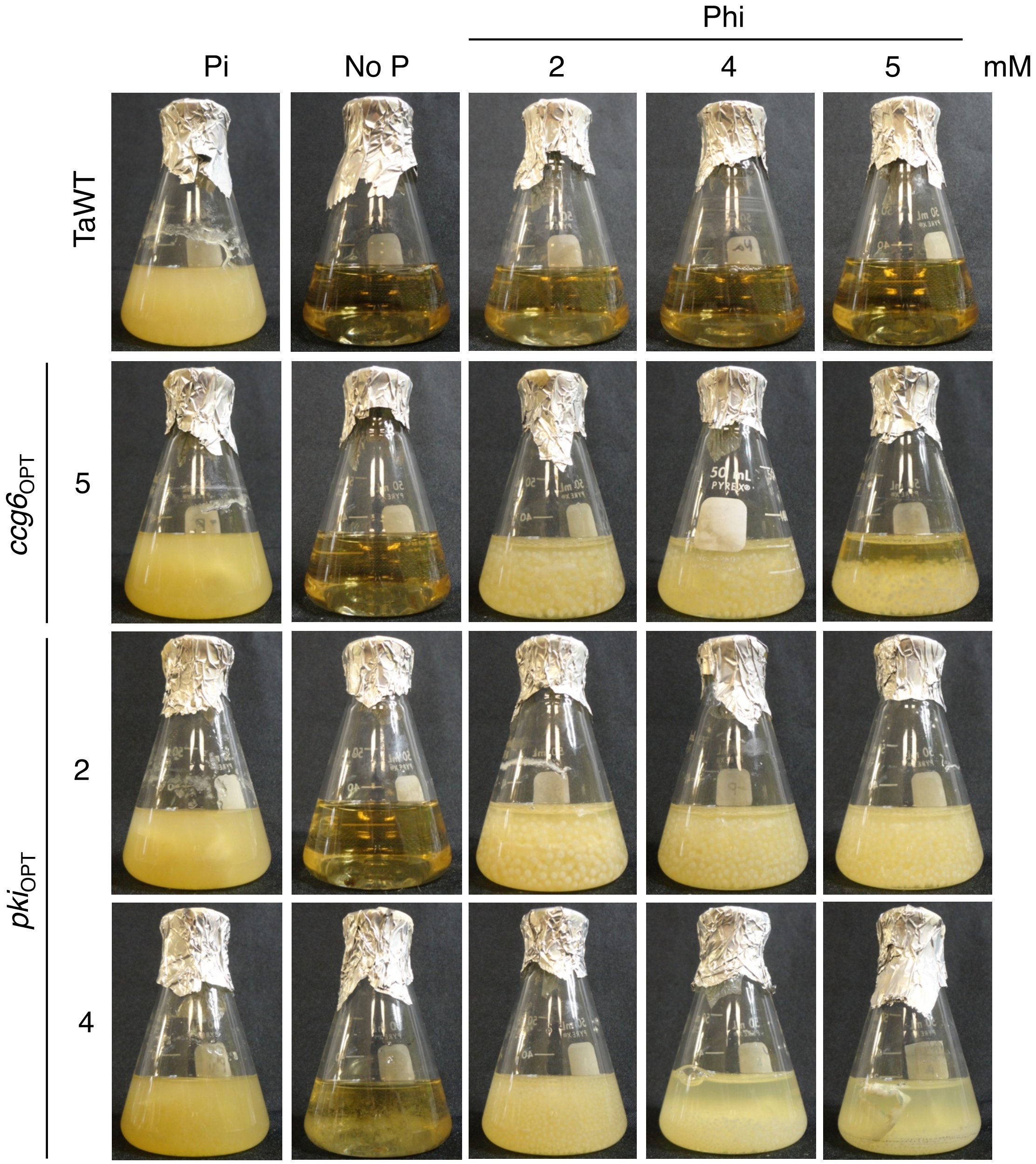

Figure S6. Growth of *T. atroviride* transgenic lines in high concentrations of phosphite. *T. atroviride* IMI 206040 (TaWT) and *T. atroviride ccg6*OPT-5 and *pki1*OPT-2 and -4 were grown in liquid Vogel's minimal media supplemented with 2, 4, and 5 mM phosphite (Phi) as the only phosphorus (P) source. Media without P (No P) and with phosphate (Pi) as the P source were used as controls. Cultures were photographed after 7 days of incubation.

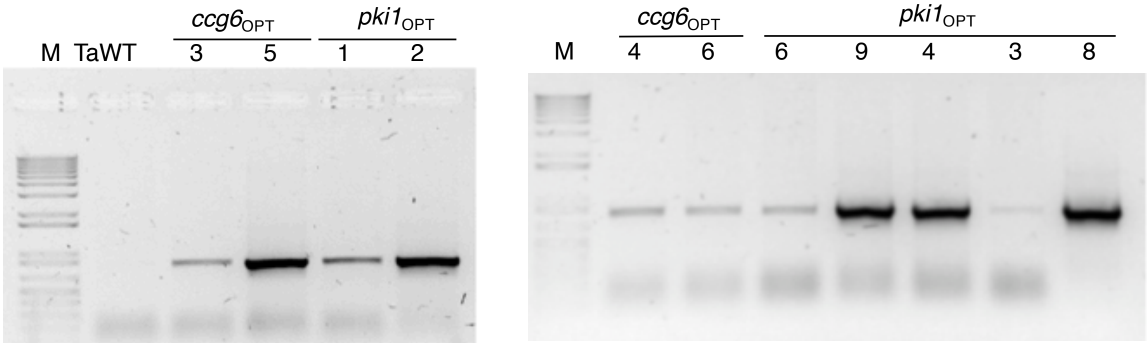

Figure S7. Detection of the *ptxD* gene in *T. atroviride* transgenic lines by PCR. *ptxD* gene was detected by PCR in different transgenic lines ($ccg6_{OPT}$ and $pki1_{OPT}$) using genomic DNA and specific oligonucleotides to amplify 840 bp of the gene. M: 1 kb plus DNA ladder. TaWT: *Trichoderma atroviride* IMI206040.

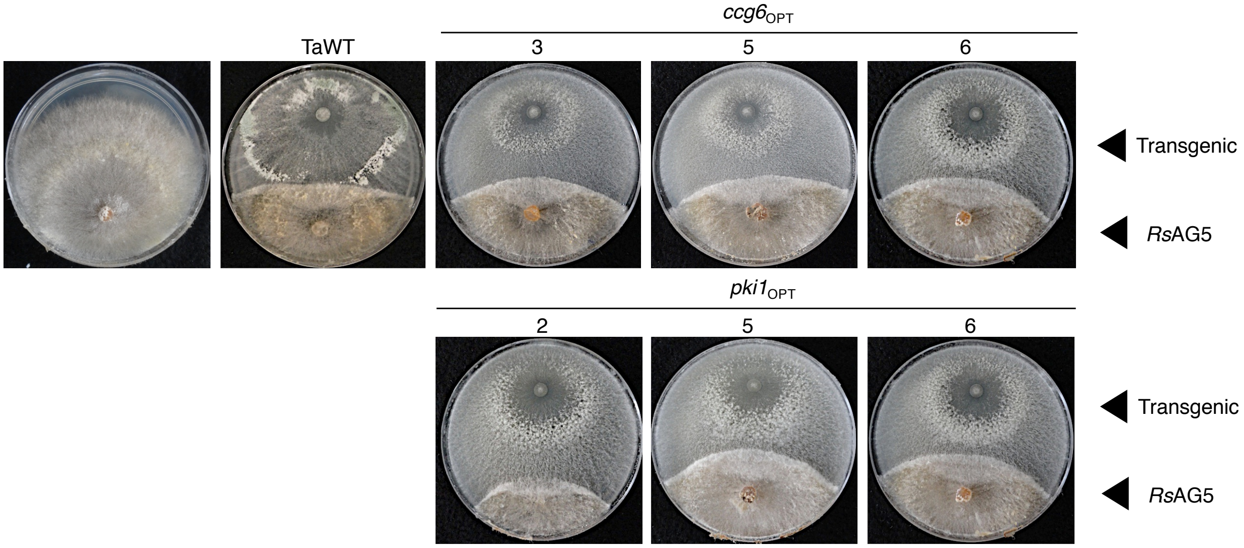

Figure S8. Confrontation experiments between the transgenic lines and *Rhizoctonia solani* AG5. Mycoparasitism activity of *T. atroviride* transgenic strains *ccg6*OPT-3, -5, -6 and *pki1*OPT-2, -5, -6 against *Rhizoctonia solani* AG5 (*Rs*AG5) grown in standard solid PDA media. *Rs*AG5 was inoculated on one side of the Petri dish and on the other the TaWT or the transgenic strains. *T. atroviride* IMI206040 (*Ta*WT) against *Rs*AG5, and *Rs*AG5 growth were used as references.

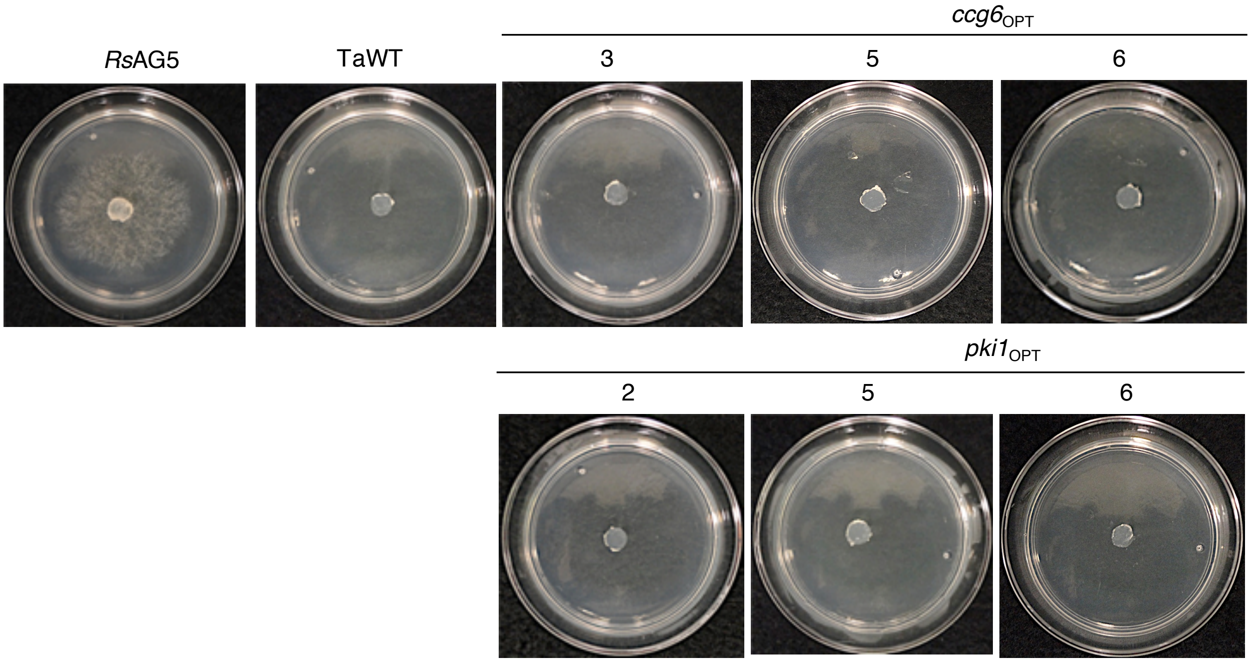

Figure S9. Antibiosis assays with Trichoderma transgenic lines. Antagonism activity of *T. atroviride* transgenic strains *ccg6*OPT-3, -5, -6 and *pki1*OPT-2, -5, -6 against *Rhizoctonia solani* AG5 (*Rs*AG5) grown in standard solid PDA media. *T. atroviride* IMI206040 (TaWT) against *Rs*AG5, and *Rs*AG5 growth were used as references.
