# Supplementary material for: Assessment of the ptxD gene as a growth and selective marker in Trichoderma atroviride using Pccg6, a novel constitutive promoter
Source: Microb Cell Fact. 2020 Mar 18;19:69. doi: 10.1186/s12934-020-01326-z (PMC7081547; doi:10.1186/s12934-020-01326-z)
Supplement: Supplementary file 2 — Additional file 2. Additional tables and legends supporting the results described in the text. [file 12934_2020_1326_MOESM2_ESM.pdf]

Assessment of the *ptxD* gene as a growth and selective marker in *Trichoderma atroviride* using P*ccg6*, a novel constitutive promoter

Nohemí Carreras-Villaseñor[1,+], José Guillermo Rico-Ruiz[1,§], Ricardo M. Chávez Montes[3], Lenin Yong-Villalobos[3], José Fabricio López-Hernández[2,†], Pedro Martínez-Hernández[2], Luis Herrera-Estrella[2,3], Alfredo Herrera-Estrella[2], Damar López-Arredondo[1,3,*]

[1]StelaGenomics México, S de RL de CV, Av. Camino Real de Guanajuato s/n, 36821. Irapuato, Guanajuato, Mexico.

[2]Laboratorio Nacional de Genómica para la Biodiversidad, Unidad de Genómica Avanzada del Centro de Investigación y de Estudios Avanzados del Instituto Politécnico Nacional, Km 9.6 carretera Irapuato León, 36500. Irapuato, Guanajuato, Mexico.

[3]Institute of Genomics for Crop Abiotic Stress Tolerance, Texas Tech University. 79409, Lubbock, Texas, USA.

*For correspondence. Email: Damar.Lopez-Arredondo@ttu.edu; Tel. 8068343364, orcid: https://orcid.org/0000-0001-7389-3143

1

[+]Present address: Red de Estudios Moleculares Avanzados, Instituto de Ecología A.C., 91070, Xalapa, Mexico.
[§]Present address: Laboratorio Nacional de Genómica para la Biodiversidad, Unidad de Genómica Avanzada del Centro de Investigación y de Estudios Avanzados del Instituto Politécnico Nacional, Km 9.6 carretera Irapuato León, 36500, Irapuato, Mexico.
[†]Present address: Stowers Institute for Medical Research, Kansas City, MO, 64110, USA.

Table S1. Colony area (cm^2) of *Rhizoctonia solani* AG5 (*Rs*AG5) and *Trichoderma* during the confrontation experiments.

Strain		Area	
		Trichoderma	*Rs*AG5
Confrontation (vs *Rs*AG5)			
TaWT		37.27 ± 0.97	19.74 ± 0.70
ccg6$_{\mathrm{OPT}}$-	3	36.66 ± 0.94	21.21 ± 0.59
	5	37.89 ± 0.66	20.50 ± 0.60
	6	37.65 ± 0.89	20.03 ± 0.40
pki1$_{\mathrm{OPT}}$-	2	36.26 ± 0.64	21.10 ±0.40
	5	37.06 ± 0.43	20.25 ± 0.52
	6	37.11 ± 0.54	19.68 ± 0.39
	8	38.29 ± 0.47	20.50 ± 0.26
Control			
*Rs*AG5		50.38 ± 0.96*****	
TaWT		58.14 ± 0.39*****	

T. atroviride IMI 206040 (TaWT) was used as positive control for confrontation tests. The values are indicated as the mean value ± SE (n=3).

*Rs*AG5 and TaWT were cultured in standard conditions as control to verify the viability of the inoculum.

*****significant at $p< 0.00001$; ANOVA-Tukey HSD.

Table S2. Colony area (cm^2) of *Rhizoctonia solani* AG5 (*Rs*AG5) and *Trichoderma* after the antibiosis test.

Strain		Area
TaWT		0.251 ± 0.009
*ccg6*OPT-	3	0.238 ± 0.003
	4	0.243 ± 0.007
	5	0.276 ± 0.023
	6	0.280 ± 0.041
*pki1*OPT-	2	0.269 ± 0.018
	4	0.232 ± 0.001
	5	0.286 ± 0.017
	6	0.231 ± 0.015
Control		
*Rs*AG5		8.589 ± 0.215

T. atroviride IMI 206040 (TaWT) was used as positive control for confrontation tests. The values are indicated as the mean value ± SE (n=3, p<0.05).

*Rs*AG5 was cultured in standard conditions as control to verify the viability of the inoculum.

Colony area included the spot where the inoculum was placed.
